# High Molecular Diversity of *Mycobacterium avium* subsp. *paratuberculosis* in Germany Revealed by Multitarget Genotyping

**DOI:** 10.3390/ijms26115273

**Published:** 2025-05-30

**Authors:** Petra Möbius, Marian Price-Carter, Heike Köhler

**Affiliations:** 1 Friedrich-Loeffler-Institut—Federal Research Institute for Animal Health (FLI), Institute of Molecular Pathogenesis, 07743 Jena, Germany; heike.koehler@fli.de; 2 AgResearch Ltd., Hopkin Research Institute, Palmerston North 442, New Zealand; marian.price-carter@agresearch.co.nz

**Keywords:** *Map*, pathogen diversity, multiple host species, multi-target-genotyping, co-infections, homoplasy, cluster analysis, epidemiology

## Abstract

This study investigated the genetic diversity of *Mycobacterium avium* subsp. *paratuberculosis* (*Map*)—the causative agent of paratuberculosis—isolated from different host species in Germany. A total of 500 isolates from 243 cattle herds and 9 other host species originating from 13 federal states were genotyped. A multi-target approach was applied, comprising IS*900*-RFLP with *Bst*EII and *Pst*I digestion; MIRU-VNTR; and SSR1, SSR8, and SSR9 analysis. In total, 93 combined genotypes were identified, 84 in cattle and 21 in non-cattle isolates. Ninety genotypes were assigned to the C-type group, and three genotypes (three from sheep and one from cattle) were assigned to the S-type/subtype III group. Cluster analysis divided genotypes into subgroups similar to those shown for WGS-SNP-based phylogenetic trees. New genotypes were revealed, including INMV262–267 and a specific sequence at locus VNTR7. Five genotypes that were predominant in cattle were also detected in sheep, goats, and deer. The majority of genotypes [61%] were identified only once. Polyclonal infections were observed in individual animals and herds, and various potential *Map* transmission linkages were uncovered. This high genotype richness of *Map* reflects the long history of paratuberculosis in Germany and intensive nationwide animal movement and international trading activity.

## 1. Introduction

*Mycobacterium avium* subsp. *paratuberculosis* (*Map*)—a member of non-tuberculous mycobacteria (NTM)—is the causative agent of paratuberculosis or Johne’s disease, a chronic infectious granulomatous enteritis with protein-losing enteropathy and severe weight loss, primarily affecting ruminants [1,2]. The disease is widespread worldwide, especially in farmed animals, but also in wild animals, and causes considerable economic losses to the livestock industries [3,4,5]. As in many other countries of the world, in Germany, paratuberculosis affects dairy and beef cattle, sheep, goats, feral red deer, fallow deer, and roe deer, as well as exotic ruminants held in zoological gardens or under private ownership [3,6,7,8,9,10]. *Map* has also been detected in some non-ruminant species, including humans [11]. A possible role of *Map* as a zoonotic agent with an etiological relevance for Crohn’s disease in humans is under longstanding discussion [12,13,14,15]. Paratuberculosis became a notifiable disease in Germany in 1970.

*Map* is an evolutionary young organism of clonal nature with a highly monomorphic genome [16,17]. Based on differences in genotype, genome structure, growth characteristics, and pathogenesis, *Map* is divided into two major phylogenetic lineages, C (cattle)-type and S (sheep)-type [18,19,20]. *Map* C-type strains, also designated as subtype II strains, have a broad host range. Although initially isolated almost exclusively from cattle, they have since been isolated from many other ruminants and non-ruminants, including humans [20]. The S-type group consists of subtypes I and III [16,21,22]. Early isolates of subtype I were mostly from sheep and also from goat [18], but more recent systematic surveys have revealed that in regions where beef cattle, deer, and sheep were held in close contact, subtype I strains were also isolated from beef, dairy cattle, and deer [23]. Subtype III strains were isolated from cattle, sheep, goats, deer, and camels [24,25,26,27]. Although there is some evidence for C-type and S-type lineage-specific differences in virulence [28], there is not yet any information about differences in the virulence of strains within the main C-type or the S-type lineages in ruminants [29]. However, there is evidence for S-type sublineage-specific variants of genes associated with virulence in mycobacteria [30,31]. Monitoring and investigation of *Map* genotype distribution, diversity, and evolution is crucial for understanding how this organism spreads, for identifying mutations that facilitate enhanced virulence and/or a broadened host range, and for finding effective new control strategies [32].

The diversity of *Map* has been extensively investigated over the last three decades using assays that target different regions in the genome [11,33,34,35]. Early studies involved one or two methods, targeting *Map*-specific or nonspecific genomic regions that were applied to panels of isolates from different hosts from many different countries worldwide [22,23,26,36,37,38,39,40,41,42,43,44,45,46], including some regions in Germany [47,48,49]. The discriminatory power of *Map* genotyping was significantly increased by combining two [22,23,48,50,51] or three complementary typing techniques [39,52,53], and along with additional epidemiological data, it was possible to uncover epidemiological links between *Map* strains originating from cattle herds and feral red deer in a National Park in Germany [8].

In 2009, an overview of *Map* genotypes originating from different hosts from seven European countries (not including Germany) was presented using a combination of pulsed field gel electrophoresis (PFGE), restriction-fragment length-polymorphism analysis based on the insertion sequence IS*900* (IS*900*-RFLP), and mycobacterial interspersed repetitive units-variable-number tandem repeat (MIRU-VNTR) typing [53]. Despite the limited number of strains per country, this overview was the first major step toward determining the level of diversity of *Map* within Europe. More recently, single-nucleotide polymorphism (SNP) analysis based on whole-genome sequence (WGS) data was used to determine the diversity of strains from different hosts of 17 regions or countries worldwide [16]. SNP analyses allow a very sensitive differentiation of isolates, the calculation of reliable phylogenetic relationships, and the tracking of putative *Map* transmission between herds and regions [16,17,54,55,56]. On the other hand, a previous study revealed only a very low number of SNPs within a panel of C-type strains from Australia and worldwide [57]. The results of WGS-based SNP analysis are very comprehensive, but the bioinformatic tools required are complex and have not been standardized. The method has thus not yet completely replaced conventional genotyping.

The purpose of this study was to investigate the molecular diversity of *Map* across Germany, to examine the distribution of identified genome variations (genotypes) among different cattle herds and different hosts and federal states, and to compare German genotyping data with that of existing data from other countries. A total of 518 isolates from cattle and nine other hosts from different herds and sources in Germany, as well as from tissue and fecal samples from individual animals, were genotyped using a combination of IS*900*-RFLP, MIRU-VNTR, and short-sequence-repeat (SSR) analysis. This study provides a comprehensive overview of combined *Map* genotypes in Germany, their similarity and presumed relationship, information about the most prevalent genotypes and possible inter-herd and inter-species transmissions, and the tendency for co-infections of individual animals with different *Map* strains.

## 2. Results

### 2.1. Genotypes Identified, Origin, and Subdivision of C- and S-Type Strains

**Figure 1:**
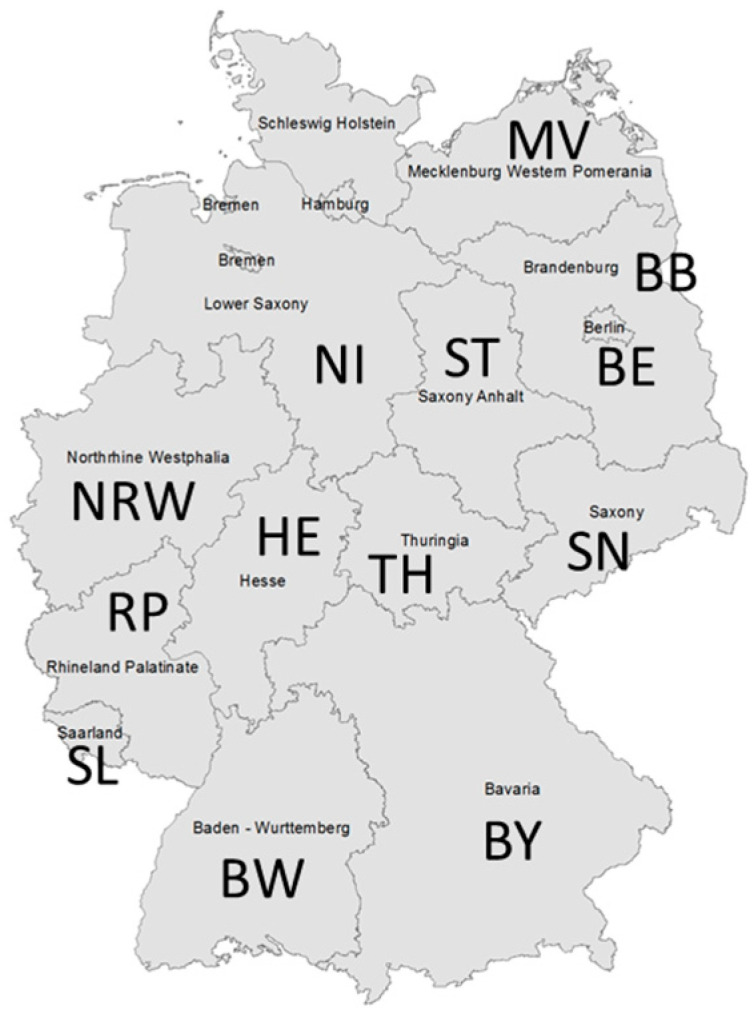
Federal states in Germany (n = 13), which were the origin of *Map* isolates characterized for this work. The number of isolates is given in brackets: BB—Brandenburg (n = 21), BE—Berlin (n = 1), BW—Baden-Wuerttemberg (n = 20), BY—Bavaria (n = 11), HE—Hesse (n = 36), MV—Mecklenburg-Western Pomerania (n = 19), NI—Lower Saxony (n = 36), NRW—North Rhine-Westphalia (n = 95), RP—Rhineland Palatinate (n = 70), SL—Saarland (n = 6), SN—Saxony (n = 51), ST—Saxony-Anhalt (n = 29), and TH—Thuringia (n = 123).

A total of 518 *Map* isolates originating from 243 cattle herds and 25 regional localities (non-cattle isolates) in 13 of the 16 federal states in Germany (Figure 1) were characterized and resulted in 93 combined genotypes (GTs), (Table 1). The results of all included isolates are listed in Table S1 (cattle isolates), Table S2 (non-cattle isolates), and Table S3 (isolates from different tissues of individual animals). Counting only the isolates with different genotypes from one cattle herd or one locality, 311 strains from cattle and 32 strains from other species, i.e., a total of 343 strains from 268 sources, were assessed as independent in this study.

The majority of the investigated isolates belonged to the C-type group and could be divided into 90 GTs. Eleven isolates were identified as S-type strains of subtype III with three GTs; nine of them originated from sheep (two flocks and two migrating herds) and two from cattle (two herds). Cattle and non-cattle hosts shared 13 GTs. A total of 57 out of 93 GTs (61%) were detected only once, including 9 GTs that were not found in cattle but were detected in a human, a donkey, goats, deer, and sheep.

### 2.2. Individual Genotypes and Discriminatory Power of RFLP, MIRU-VNTR, and SSR Typing

#### 2.2.1. Previously Detected and Novel Genotypes

**Figure 2:**
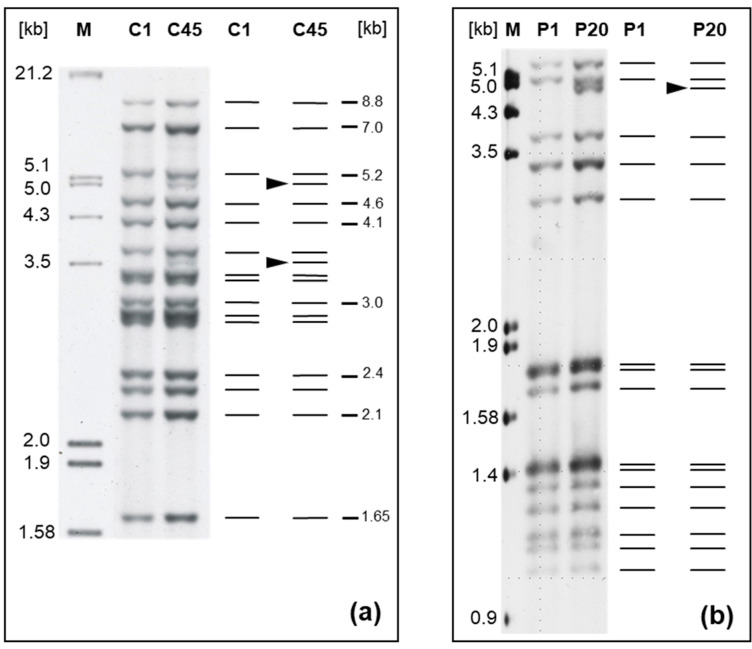
New IS*900*-RFLP profiles of *M. avium* subsp. *paratuberculosis* strains from cattle. (**a**) Profile C45 (strain 06A0169) compared to reference profile C1 (strain 06A0168) after chromosomal DNA digestion with *Bst*EII and (**b**) profile P20 (strain 12MA1661) compared to reference profile P1 (strain 12MA1602) after chromosomal DNA digestion with *Pst*I. A digoxigenin-labeled specific 453 bp fragment of IS*900* was used as probe. The numbers above the lanes are the type designations. The black arrowhead in the schematic diagram shows the difference between type C1 [58] and novel type C45, as well as P1 designated according to [59] and novel type P20. Lane M represents the molecular weight marker III (Roche Diagnostics). Numbers on the right in (**a**) indicate sizes of reference bands.

The 93 GTs consisted of 39 IS*900*-RFLP patterns (a combination of 26 and 17 band patterns after *Bst*EII and *Pst*I digestion, respectively), 33 MIRU-VNTR, and 15 SSR profiles. Two novel IS*900*-RFLP patterns (C45 and P20; see Figure 2) and seven novel INMV profiles (Table 2) were discovered. All IS*900*-RFLP band patterns are shown as schematic representations and corresponding gel images of Southern blots in Figures S1–S3.

#### 2.2.2. Specific Sequence Pattern at VNTR7

The new MIRU-VNTR profiles include INMV30* (325223*28) with a new sequence pattern at locus 7, identified by sequencing and in silico analysis: in addition to two complete copies of VNTR7 repeat (CGAAATATTCGCCGTGAGAACA), a different sequence of 24 nucleotides was detected. This consisted of an additional imperfect back-flanking sequence region of 12 nucleotides (nt)—(CGTGCGGCGAAG)—followed by an imperfect repeat of 12 nt (GCCGTGAGAACA), (plus CG). The amplicon size appeared like three repeats, and the number of repeats was therefore designated as 3*. Profile INMV30* was found in two isolates from intestinal lymph nodes of two individual cattle in a single herd in Thuringia (Table S1).

#### 2.2.3. Number and Frequency of Individual IS*900*-RFLP, MIRU-VNTR, and SSR Types

In the following text, the number n (in brackets) represents the number of independent strains originating from different herds or sources and belonging to the individual genotype of the respective typing method.

Typing by IS*900*-RFLP with *Bst*EII digestion revealed 303 independent strains with the following 4 of 26 patterns identified most frequently: C1 (n = 202, 67%), C17 (n = 22, 7%), C18, and C41 (n = 17, 6%). Typing via IS*900*-RFLP with *Pst*I digestion revealed 302 independent strains, and 5 of the 17 detected patterns were most often found: P1 (n = 184, 61%), P3 (n = 36, 12%), P16 (n = 16, 5.3%), P7, and P8 (n = 15, 5%). IS*900*-RFLP typing after *Bst*EII and *Pst*I digestions resulted in 39 combined profiles and 313 independent strains. Type C1-P1 (n = 174, 56%) and C1-P3 (n = 31, 10%) were most frequently detected. A total of 14 profiles were found in multiple (2 to 16) strains, and 23 profiles were found only once. However, the most common IS*900*-RFLP (*Bst*EII) C1 type was subdivided into 42 combined genotypes in this study.

Using MIRU-VNTR typing, 318 independent strains could be differentiated, exhibiting the following 4 of 33 profiles most frequently: INMV2 (n = 95, 30%), INMV1 (n = 58, 18%), INMV19 (n = 38, 12%), and INMV33 (n = 37, 12%).

Finally, SSR typing revealed 299 independent strains, and the following 3 of 15 profiles were detected most frequently: 7-4-4 (n = 198, 66%), 7-4-5 (n = 39, 13%), and 7-5-5 (n = 20, 7%). The results for each individual genotype identified using the different methods and the number of independent strains with that genotype are listed in Table S4.

#### 2.2.4. Discriminatory Power of Typing

Based on the total of 93 identified genotypes and the resulting 343 independent strains, the discriminatory power of the combined genotyping technique was calculated and showed a DI value of 0.954. IS*900*-RFLP (*Bst*EII, *Pst*I), MIRU-VNTR, and SSR typing each had a lower discriminatory power, with DI values of 0.67, 0.847, and 0.54, respectively (Table S4).

### 2.3. Comparison of Combined Genotypes and Phylogeny

**Figure 3:**
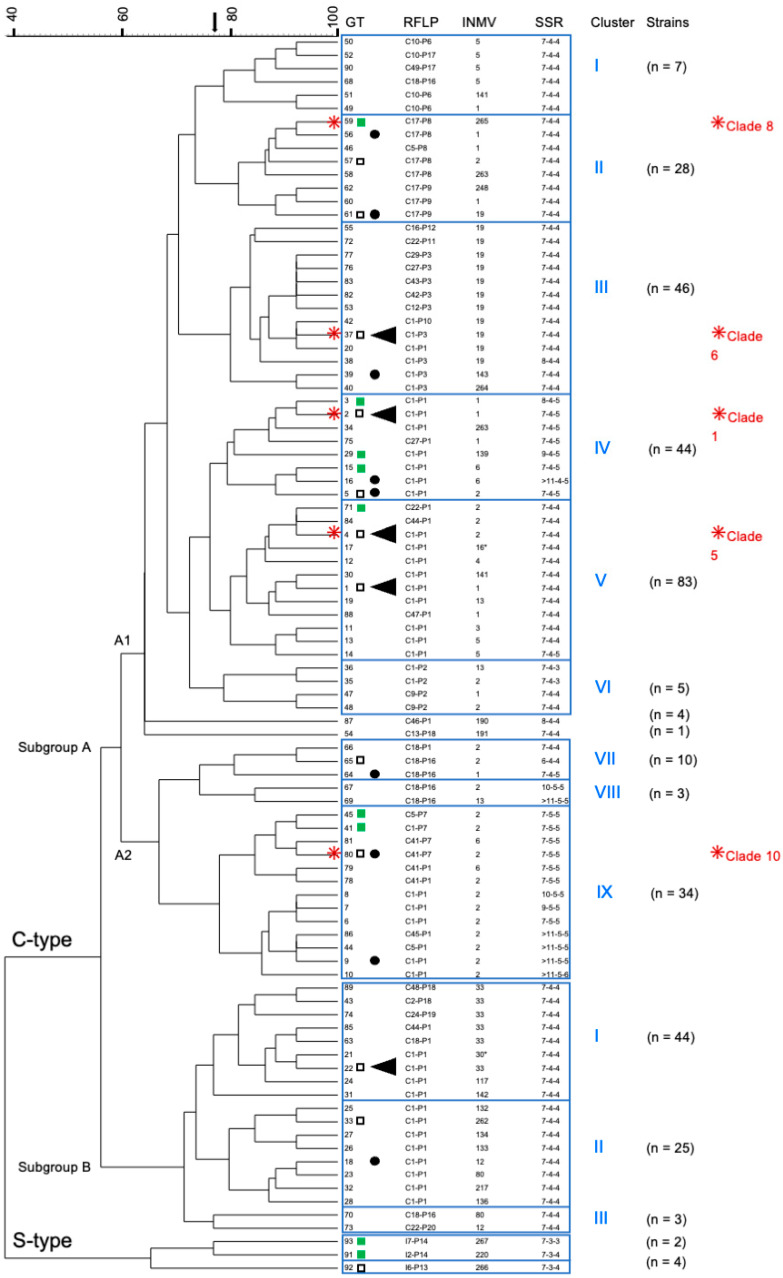
This dendrogram (UPGMA-tree) shows the similarity of 93 combined genotypes (GTs) of German *M. avium* subsp. *paratuberculosis* strains and their assignment to main groups, subgroups, and clusters. The tree was calculated based on the results of IS*900*-RFLP (*Bst*EII, *Pst*I), MIRU-VNTR typing (8 loci; only INMV profiles are shown), and SSR analysis (SSR1, 8 and 9). The five most frequently detected GTs 1, 2, 4, 22, and 37 (found in 16–52 independent strains) are highlighted by (◄), and frequently detected GTs (found in 7–14 independent strains) are indicated by (●). The number n of independent strains that belong to the respective clusters is given in parentheses. (□) GT was determined in cattle as well as non-cattle isolates. (■) GT was determined in non-cattle isolates only. All other GTs have so far only been found in cattle isolates. 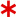 GTs of five German isolates included in a previous WGS-based SNP analysis [16] and a SNP-based assay with assignment to specific phylogenetic clades [60] are shown with the respective clade number in red letters.

The relationship of the identified genotypes is shown in Figure 3. The 93 genotypes were grouped into C-type and S-type, with C-type further divided into the main subgroups A and B, with nine and three clusters, respectively. German strains with genotypes GT59, 37, 2, 4, and 80 (Figure 3), assigned to five clusters within Subgroup A, were included in two previous WGS studies [16,60], where they were assigned in a SNP-based phylogenetic tree to five clusters within Subgroup A of the C-type group [60].

### 2.4. Distribution of Map Genotypes Among Different Herds, Hosts, and Federal States

**Figure 4:**
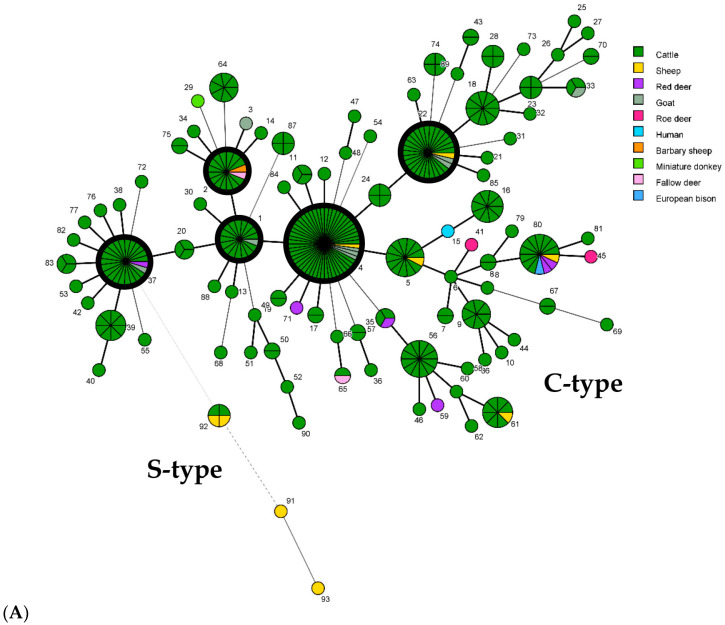

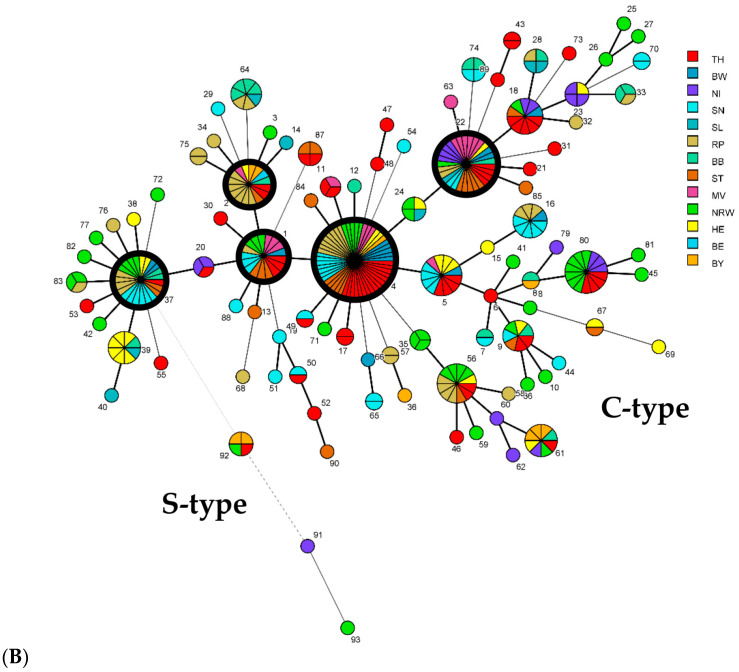
Minimum spanning tree (MST) based on comparison of 93 combined genotypes of *M. avium* subsp. *paratuberculosis* isolates identified in this study. Each genotype (GT) is displayed as a node, dimensioned proportional to the number of clustered independent strains harboring this specific GT (node numbering = GT, according to Table 1). A combined genotype from each herd/local source was considered only once, regardless of the number of isolates with that GT. In total, the GTs of 343 independent strains from cattle of 243 cattle herds and 32 independent strains from 9 other hosts in 25 other sources in Germany were included. Thick black-bordered circles represent the five most common GTs (GT1, 2, 4, 22, and 37). The distances between nodes (corresponding to GTs) vary depending on the number of typing target regions that differ. Thick lines between two nodes represent a difference at one target region, and thin and dashed lines show a difference at two or more typing targets. (**A**) Different colors highlight the different host origins of strains. (**B**) Different colors represent the origin of isolates from the different federal states in Germany (see Figure 1).

The minimum spanning trees (Figure 4) show the distribution of genotypes among herds/sources, hosts, and federal states. No correlations were found between geographic origins (federal states) and the predominant GTs.

The number of cattle herds, tested isolates, and identified GTs per federal state is summarized in Table S5. In addition, the origin of each combined genotype is listed in Table S6.

### 2.5. Diversity of Isolates

Altogether, 84 GTs were detected in cattle (83 C-type and 1 S-type), with 49 GTs unique to single herds across all federal states, affecting 20% of herds. A total of 30 GTs were found in 2 to 12 cattle herds and in up to six federal states (Figure 4), and 5 predominant GTs were detected in 14 to 48 cattle herds and in up to 10 federal states (Table 3).

Non-cattle isolates exhibited 18 C-type and 3 S-type GTs (Table 1, Tables S2 and S7), with 7 C-type and 2 S-type GTs unique to non-cattle. Predominant cattle GTs were also found in sheep (GT2, 4 and 22), goats (GT1, 4, 22, and 37), and deer (GT2 and 37). Ten GTs (all C-type) were detected in isolates from wild living ruminants (red deer, roe deer, fallow deer, and European mouflon), with four unique to them.

### 2.6. Intra-Herd Diversity of Map

Multiple *Map* isolates were characterized from 70 herds, detecting one genotype in 22 herds and multiple genotypes in 48 herds, regardless of whether isolates were collected at the same time or at intervals of one to ten years (Table S8). In herds with multiple GTs, two to eight GTs per herd were found. Among these, there were herds in which one genotype predominated (NRW-4) and remained detectable over several years (TH-1, RP-12, and RP-14; see Tables S1 and S8).

In 36 herds, GTs differed at 2 to 8 of the 13 target regions for typing. The remaining 12 herds harbored strains that differed at only one target region (Table S8).

The intra-herd strain type diversity of *Map* varied. Diversity was calculated for two herds with more than 20 isolates genotyped and was higher in herd NRW-12 (23 isolates with eight GTs) than in herd TH-1 (28 isolates with 4 GTs), exhibiting a Shannon diversity index H = 1.514 versus H = 0.559 and a Simpson index D = 0.265 versus D = 0.733 (Table S9).

### 2.7. Mixed Genotypes and Co-Infection of Individual Animals

#### 2.7.1. Mixed Genotypes in Individual Isolates

Double PCR-bands observed in the agarose gels at one or two specific MIRU-VNTR loci indicated mixed genotypes in ten cases (Table 4). In three cases, multiple target regions showed genotype differences. The suggested GTs were confirmed in several cases by separate detection of one or both GTs in the same herd (Table S1). All mixed GTs were found in cattle isolates from fecal and intestinal content and boot swab samples (Table S1).

#### 2.7.2. Different Genotypes in Isolates from Individual Animals

Two different GTs were identified per animal in isolates from different tissues and fecal samples of 9 out of 22 animals (Table 5 and Table S3).

In herd TH-1, GT61 was predominant in 24 out of 25 cattle (Table 5 and Table S1), with 4 animals showing a second GT differing in one to three target regions (Table 5). One sampled sheep (animal 9) was co-infected with S-type and C-type strains detected on different media.

### 2.8. Suspected Epidemiological Linkages

**Figure 5:**
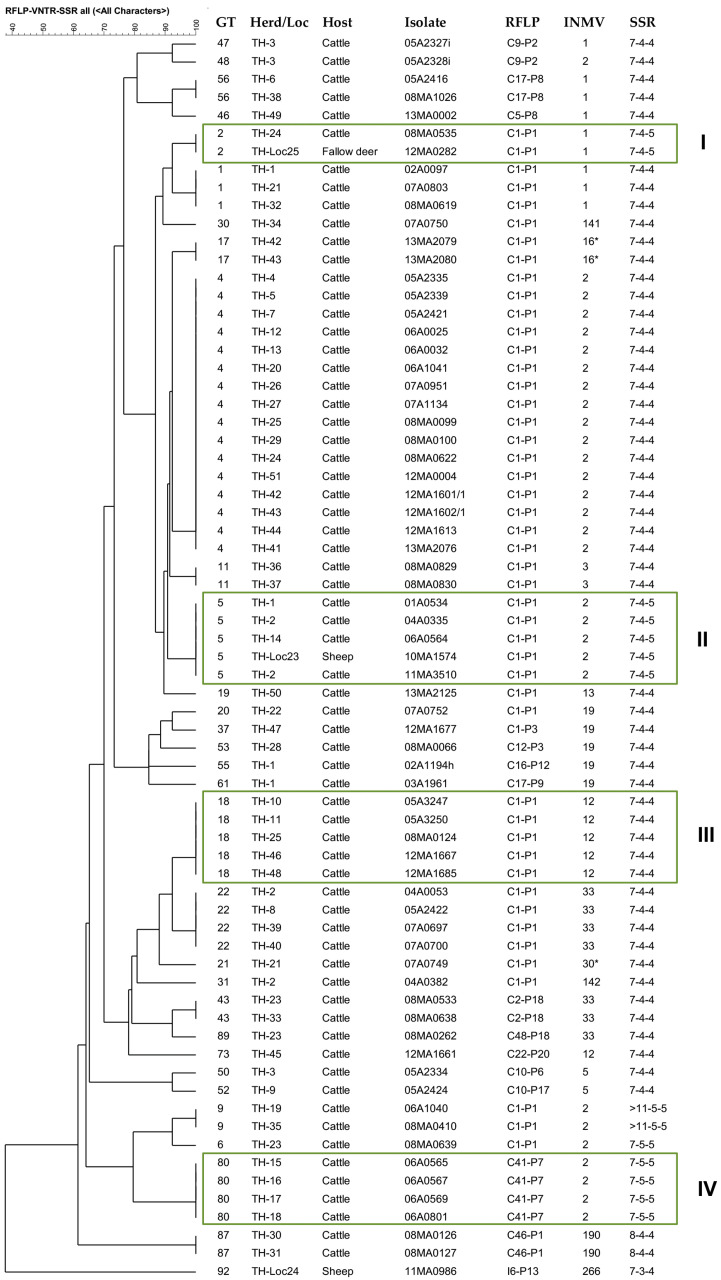
IS*900*-RFLP/MIRU-VNTR/SSR based tree of similarity (UPGMA) of 68 independent *M*. *avium* subsp. *paratuberculosis* strains with 31 genotypes across 51 cattle herds, 2 fallow deer, and 2 sheep (including one European mouflon) in Thuringia. Identical genotypes (GTs) suggest possible epidemiological links: Boxes I and II show isolates with identical GT originating from different hosts; boxes III and IV include identical GTs found in isolates from cattle of 5 or 4 herds, respectively. * INMV16* (323325*28) is a profile with an altered repeat sequence at VNTR locus 7.

**Figure 6:**
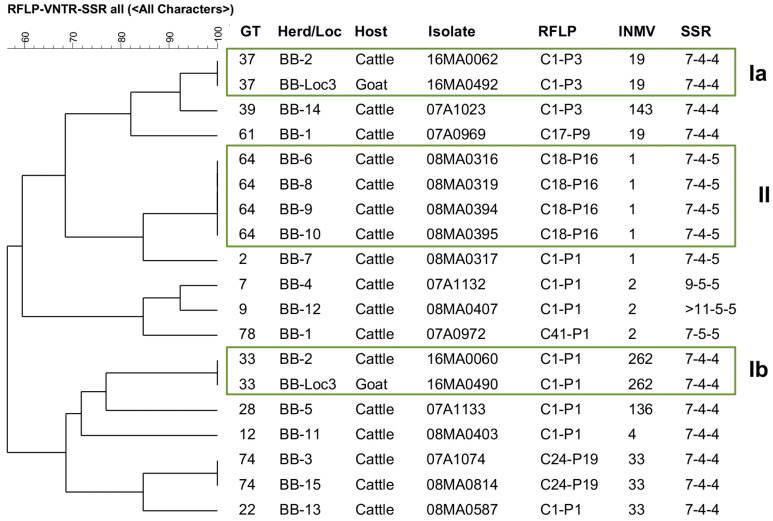
IS*900*-RFLP/MIRU-VNTR/SSR based tree of similarity (UPGMA) of 17 *M. avium* subsp. *paratuberculosis* strains from cattle and 2 from goat originating altogether from 15 herds in Brandenburg. Only cattle isolates with different genotypes (GT) from the same herd (independent strains) were included. Boxes Ia and Ib highlight two GTs identified in isolates from cattle and goats from the same herd (BB-2 = BB-Loc3). Box II shows identical GT found in different cattle herds.

Infection with the same strain was assumed if isolates from a single animal, herd, or other source had the same GT. Shared GTs among different herds or hosts suggested possible epidemiological linkages (Figure 4). Figure 5 and Figure 6 illustrate the genotype distribution in Thuringia and Brandenburg.

In Thuringia (TH), the predominant GT4, GT22, and GT1 suggest links within TH, but also to herds in other federal states. GT2, identified in a cow and fallow deer (Box I), and GT5, found in cattle and a sheep (European mouflon) (Box II), indicate possible interspecies transmission. GT5 was confirmed in isolates of a herd from 2004 to 2011, indicating the persistence of a genotype in a herd over a longer period of time.

In Brandenburg, different GTs were identified in a cattle herd and in a herd keeping cattle and goats (BB-2 = BB-Loc3). The goats were introduced to this herd in 2011 and were temporarily grazed together with cattle on the same pasture. At the time of strain isolation (2016), cattle and goats were suffering from clinical paratuberculosis. Two identical GTs in both species suggest interspecies transmission in this herd. In addition, GT64 was found in four cattle herds, which could indicate transmission by animal movement.

## 3. Discussion

Paratuberculosis has long been recognized and is widespread worldwide [3,61,62,63]. The high diversity of *Map* revealed in this study reflects, to some extent, the long history of this disease in Germany, including the intensive trading activity within the country and with neighboring countries in Europe (ec.europa.eu/Eurostat/data), as well as direct or indirect *Map* transmission between livestock and wild living animals [8,41].

The current study began before the WGS-based SNP analysis for genotyping *Map* was introduced. IS*900*-RFLP typing was carried out in the laboratory initially, followed by MIRU-VNTR and SSR analyses. Like other fingerprinting methods, IS*900*-RFLP is a technically demanding and time-consuming laboratory procedure and was replaced by MIRU-VNTR, multi-locus SSR, and WGS-based SNP analysis in several subsequent studies. Nevertheless, this study presents gel images and schematic overviews of the IS*900*-RFLP profiles based on *Bst*EII and *Pst*I digestion of German isolates and combines IS*900*-RFLP with MIRU-VNTR and SSR profiles for individual strains.

### 3.1. Genotyping Markers and Homoplasy

In general, strain diversity is caused by the plasticity of the *Map* genomes, which change slowly relative to many other bacterial genera through spontaneous mutations and as a result of changing selective pressures in their microenvironment [20]. Natural events such as recombination and slipped-strand mispairing during replication lead to VNTRs and SSRs [64] with varied repeat numbers, possibly facilitating reversible shutdown or modulation of the function of specific bacterial genes [65,66]. These changes are in addition to major differences between C- and S-type with gene insertions and deletions and differing genome structure [31,67,68]. *Map* strains within the main lineages differ genetically in their content of single-nucleotide variants (SNVs) or SNPs [16,17,31,69], indels, and repeat sequences [22,46], as summarized in [20]. Finally, these polymorphisms cause different IS*900*-RFLP patterns (also based on SNVs or SNPs at restriction sites of digestion enzymes) and different MIRU-VNTR and SSR profiles (based on repetitive mini- and microsatellites) used in this study for genotyping.

The genetic markers of genotyping have varying mutation rates [50,70,71]. SNPs are very stable and therefore useful for phylogenetic studies [16], while MIRU-VNTR loci and SSRs may be subject to homoplasy in *Map* genomes, affecting phylogenetic accuracy [16,55,56,71]. MIRU-VNTR typing results were found to both over- and underestimate the phylogenetic relationships between strains compared to WGS-SNP analysis results [16,54,55,56,71]. In addition, the results of SSR typing at 11 SSR loci did not show any significant correlation with WGS-based SNP clustering [55,56], probably caused by erroneous sequencing results at SSR1 and 2 with more than 11 mononucleotide repeats [39] and a very high mutation rate at SSR2 [8]. In this study, we excluded SSR2 and used SSR1 up to ≥11 repeats.

However, previous studies have demonstrated the sufficient stability of the genetic markers employed in the current study for revealing *Map* diversity and performing epidemiological investigations on a limited temporal and spatial scale [8,72].

### 3.2. Phylogenetic Grouping and Comparison with WGS-Based SNP Analysis

The phylogenetic relationships of *Map* strains are currently calculated using WGS-based SNP analyses of the core genomes [16,55]. However, these analyses, usually based on short-read sequence data, exclude information about genome structure, IS elements, and other repetitive (micro and minisatellite) sequences, which can be detected partly by IS*900*-RFLP, MIRU-VNTR, and SSR analyses. In the similarity tree of this study (Figure 3), the assignment of combined genotypes in the C-type group to two main subgroups, A and B, and several sub-clusters was very similar to that in two WGS-SNP-based phylogenetic analyses, including a large number of *Map* strains, originating from different hosts and regions worldwide [16,60]. As mentioned above (Section 2.3), five German strains analyzed in the present study were also included in these two previous studies. These strains were assigned to five phylogenetic clades in Subgroup A [60], which corresponds to their assignment to five sub-clusters in the similarity tree of the present study. These similarities suggest a phylogenetic relevance of the calculated relationships based on combined genotypes, despite the suspected homoplastic properties of some MIRU-VNTRs and SSRs sequences [16,17,55].

In both the similarity tree and the SNP-based phylogenetic tree [16], genotypes with INMV1 and INMV2 profiles were only assigned to subgroup A, and only Subgroup B contained strains with INMV33, INMV80, and other individual profiles. The groupings indicate a possible association between certain genomic markers/target regions of the typing approach used, such as INMV profiles, and SNP-based phylogeny, providing validation of both methods for use in classifying *Map* isolates. A strong statistical association between phylogeny and INMV1, but not INMV2, was previously revealed in another WGS-based study of 200 *Map* C-type isolates from Western France [55]. Future studies using WGS-based SNP analysis of the German *Map* strains will further clarify the phylogenetic relevance of current clustering.

### 3.3. Genotypes of Map S-Type Strains

Apart from sheep and cattle in Germany, subtype III strains have been previously detected in several countries worldwide in sheep (Canada, South Africa, Spain, and the United States), as well as in goats (Canada, Spain, Switzerland), deer (The Netherlands, Czech Republic), and cattle (Canada, Switzerland) [17,25,53,73,74], whereas subtype I strains have been mostly reported in sheep (Scotland, England, Hungary, Spain, the Faroe Islands, Morocco, South Africa, New Zealand, and Australia), but also in deer, goat, dairy, and beef cattle (Spain, Hungary, and New Zealand) [16,23,24,36,42].

In this study, three genotypes belonged to S-type/subtype III, detected in six epidemiologically independent strains. In Germany, there is a significantly lower number of sheep farms compared to cattle farms, and sheep and cattle are usually farmed separately. In addition, sheep in Germany were tested for *Map* infection less frequently than cattle, which could also explain the paucity of *Map* S-type stains in this study set. All three genotypes (GT91-93) were isolated from sheep, but GT92 was additionally isolated from two cattle herds without known contact with the two sheep herds with GT92. To the best of our knowledge, this is the first report of *Map* S-type isolated from German cattle. *Map* S-type strains have also been found occasionally in cattle in other countries, e.g., in Scotland [75], Australia, Iceland [76], Spain [77], subtype I in New Zealand [23], and subtype III in Canada [17] and Switzerland [74]. Direct or indirect contact of cattle with paratuberculosis-infected sheep was reported as a major reason for transmission of S-type strains between sheep and cattle [23,76]. No further epidemiological information was available for the *Map* S-type affected German cattle herds.

Genotype GT91 (I2-P14/INMV220/7-3-4) was isolated from a sheep flock with a chronic paratuberculosis problem. Profile INMV220 was also reported for *Map* isolates from a sheep (2016) and a goat (2018) in different cantons of Switzerland (Scherrer, personal communication, [78]). However, the VNTR profile [22331(1.5)18] in INMV220 is identical to that in INMV124, which has already been found in *Mycobacterium avium* subsp. *hominissuis* (*Mah*) isolates from pigs [79,80], a Tapir [81], a dog, a chicken, and other sources (Möbius, personal communication). At the VNTR 7 locus, all S-type strains and strains of the *Mycobacterium avium* complex (MAC) harbor 1.5 repeats [82], including *Mah* strains with profile INMV124 [79]. However, a specific SNP (C > T) at position 89 bp in the VNTR 7 locus outside the true repeat sequence(s) distinguishes *Map* from other MAC genomes [82], confirmed for *Map* S-type strains (C) in this study and for different *Mah* strains (T) with INMV124 (Möbius, personal communication).

### 3.4. Diversity of Map C-Type Strains

The majority of GTs identified in this work belonged to the C-type group (90 GTs) and were found in 507 isolates from 243 cattle herds and 22 other sources in Germany. Earlier studies by other authors on *Map* diversity in Germany, conducted from 2009 onwards, examined fewer isolates, mostly cattle isolates, from fewer sources and in only one or three federal states, and used other combinations of typing methods from those employed in the present study [47,48,49]. However, all revealed a relatively high diversity of C-type strains in Germany by applying more than one typing method. Employing our applied typing approach on such a large number of isolates from different herds and regional origins has provided an even greater estimate of diversity within the *Map* population in Germany than previously determined.

Various combinations of analysis tools have also been applied to reveal the *Map* diversity in different studies conducted in other countries. Comparing the results of typing from these previous studies to results from this study shows varying diversity of *Map* in individual regions in the middle of Europe and the detection of several dominant genotypes common to Germany and neighboring countries.

In a study by Stevenson et al. [53], the diversity of *Map* in different hosts from seven European countries (not including Germany) was presented. A total of 44 combined profiles (IS*900*-RFLP [*Bst*EII] + PFGE multiplex + MIRU-VNTR) were found in 164 isolates from 16 host types, including 15 profiles from 35 cattle and 10 profiles for 13 isolates from wild ruminants. In the present study, significantly more combined profiles (n = 84) were identified in German cattle, perhaps reflecting the higher number of cattle isolates characterized or the different typing approaches applied. There was agreement when diversity from the same methods was compared. About 70% of all isolates in the Stevenson study [53] showed IS*900*-RFLP profile C1, 11% in combination with profile INMV1, and 12% in combination with profile INMV2. In the current study, 76% of all isolates harbored IS*900*-RFLP profile C1, 10% in combination with INMV1, and 23% with INMV2.

Only 20 combined genotypes were identified in a study from Austria that employed IS*900*-RFLP [*Bst*EII, *Pst*I] and MIRU-VNTR analysis to type 249 isolates from cattle, goat, sheep, and wild ruminants from different herds and regional origins [51]. In the present study, 80 genotypes were found, based on both methods, suggesting a higher *Map* diversity in Germany than in Austria.

Of the 33 INMV profiles identified in German strains, including 29 INMV profiles from cattle, 7 profiles (INMV262–267, and 30*) have not yet been published. INMV30* and 16* with unique sequences at VNTR 7 locus did not correspond to the specifications for the MAC-INMV-SSR database [78] and were therefore probably not registered in the database, although special sequences at VNTR 7 locus were also described previously [82]. INMV2 and INMV1 were the most common profiles in Germany (30% and 18%). These were also the predominant profiles in other countries in Europe, Canada, Argentina, and New Zealand, followed by INMV19 and 33 (12% each), which were found less frequently or not at all outside of Germany [16,43,44,50,51,53,55,71,83] (Price-Carter, personal communication). Apart from the different numbers of isolates, herds, and regional origins, the total number of INMV profiles detected so far in Canada, Austria, Ireland, and Argentina was lower [44,51,71,83] than in Germany or France (this study [50,55]). Furthermore, several INMV profiles appeared more frequently in other countries, e.g., INMV3 in Canada and INMV11 in Argentina [56,83], and some were not identified in German strains.

The most common SSR typing profiles in Germany, 7-4-4, 7-4-5, and 7-5-5 (three out of 15 profiles), were also the most frequently detected in isolates from France and Ireland, 7-4-4 (combined with INMV1 and 2), 7-4-5 (combined with INMV1), and 7-5-5 (combined with INMV2) [39,40]. Other SSR profiles were predominant in strains from the United States, including profile 7-6-5 [84], which has not yet been detected in Germany, profile 7-5-5, and profile >11-5-5 [85].

The marked differences in *Map* diversity in different countries and regions of the world, discussed above and in [54], are related to the history of paratuberculosis and the intensity and direction of global cattle trade, which has increased dramatically over the last century. Differences in *Map* diversity between continental European territories and Ireland or Canada and the introduction and persistence of only subsets of genotypes in these countries were confirmed by WGS-based SNP-analysis and other bioinformatic tools [17,54].

### 3.5. Intra-Herd Diversity of Map and Co-Infections of Herds and Individual Animals

Different studies have shown that the intra-herd variation of *Map* strains depended on the frequency at which infected animals were introduced into herds from different sources [86]. The results from the current work also confirm this tendency, as a significantly higher *Map* diversity was found in a cattle herd with known intensive animal movements than in a cattle herd with one predominant infection strain without intensive cattle movement (Table S9).

Co-infections of herds or individual animals with multiple genotypes of *Map* were reported in several previous studies [49,51,55,86]. In the present study, two to eight GTs were found in 66% of tested cattle herds. In 36 of these 48 herds with multiple GTs, the polymorphisms involved two, but mostly four to eight of the 13 target regions, indicating more distantly related strains and separate infection events caused by different strains. The target sites that varied most frequently included SNPs at digestion sites of restriction enzymes responsible for different RFLP patterns, several VNTR loci (VNTR292, 7, 47, and 25) as well as SSR8 and 9. In 12 of the 48 herds with multiple GTs, the isolates varied in only one, but different target regions (without a preference of a specific genome marker), suggesting spontaneous random mutations of a *Map* strain within the affected herd rather than co-infection with different strains.

*Map* infections in wild and domestic ruminant species in general seem to be polyclonal [41]. In the current study, multiple genotypes of individual animals were identified in mixed isolates and in isolates from different tissues. In seven of ten mixed isolates, only one polymorphism was detected, again suggesting recent mutation rather than a co-infection with different strains.

After multiple infections with different strains and during different infection processes, the bacteria can be disseminated to different sections of the intestinal system and also to different extraintestinal tissues [87,88], which was also confirmed by the results of this study: *Map* was isolated also from liver, spleen, muscle, udder, and lung (and genotyped). As described above for individual herds or isolates, and also discussed previously [89], multiple genotypes found in tissue and different fecal samples of individual animals varied either in only one target region or in several, again reflecting both within-host/herd evolution and/or multiple introductions.

One sheep was even co-infected with a C-type and an S-type strain, detected by using different culture media. Systematic cultivation of isolates on different culture media could possibly reveal more polyclonal infections, as individual strains or groups favor certain ingredients, as described for *Map* C-type and S-type strains [19,90].

### 3.6. Epidemiological Findings

The results from the current multi-target study provide useful information about the *Map* distribution and possible epidemiological links of specific *Map* genotypes in Germany (see Figure 4, Figure 5 and Figure 6). The multi-target approach used in this study provided a value of >0.954 (DI) deemed sufficient for epidemiological studies [91].

The five predominant genotypes in German cattle were widespread, perhaps suggesting that present phenotypes have a similar virulence or are highly adaptable. Because these GTs were distributed across a wide range of federal states, it was not possible to draw firm conclusions regarding transmission routes via animal movements. Certain GTs occurred more frequently in individual states, but there was no statistically significant association between the genotypes and the regional origin. This lack of association could be due to a high exchange of cattle between herds in Germany, as has been shown for other European regions—for example, France [92] and the West of France [55].

Interestingly, the predominant type GT22 (including INMV33) was mostly found in the former Eastern Germany states, in Mecklenburg-Western Pomerania, Saxony-Anhalt, and Thuringia, possibly caused by the import of cattle from Lower Saxony after 1989. The rare GT17 (with INMV16*), reported for cattle isolates from Thuringia in this study, was also identified (without RFLP results) in a goat herd in TH [93], suggesting a possible epidemiological link between cattle and goats in this federal state.

Only a few isolates from wildlife and domestic non-cattle species were characterized; therefore, our study gives only limited insight into the diversity and epidemiology of *Map* in non-cattle species. The seven unique C-type GTs, not found in cattle, originated from a donkey born in France [94], a healthy human, two wild living red deer, two wild living roe deer, and a goat (from a private husbandry). The eleven other C-type GTs from non-cattle hosts were shared with cattle, including the predominant GTs as well as six others. Ten of them were found in both cattle and non-cattle in the same federal state (Table S7), suggesting interspecies transmission, as discussed in previous studies [23,37,51,53].

Ten genotypes were detected in wildlife isolates, four of which had three unique IS*900*-RFLP patterns and one new INMV profile (INMV265). This could indicate a specific *Map* population in wildlife, possibly as a result of evolution or immigration of wildlife from neighboring countries [8]. The other six GTs were shared with cattle, suggesting interspecies transmission of these strains, as described above and discussed before [8,51].

Wildlife could have become infected through shared pastures contaminated with *Map*; from farm animals or within their population at feeding sites, especially in winter; or even by contact across national borders [8,95]. Furthermore, infections can be introduced into zoological gardens through the purchase of sub-clinically infected animals and can be transmitted between zoo animals of different species through shared habitats [96].

Nearby Germany, in Austria, ten profiles were detected by MIRU-VNTR in isolates from five species of wild living ruminants [41]. Apart from INMV1 and INMV2, eight other profiles were identified that do not occur in German wildlife isolates. However, as in Germany, data from studies in Austria show that there are multiple epidemiological links between isolates from wild and farm animals [51], and some wild animals appear to be infected by specific *Map* genotypes.

The comparison of data from Austria [41,51] with data from this study did not provide evidence of *Map* transmission between wildlife from the two countries. But because there were only a small number of wildlife isolates characterized in our study, it was not possible to draw firm conclusions. Further studies are required for this purpose.

## 4. Materials and Methods

### 4.1. Map Isolates

The *Map* field isolates used in this study were collected between 1993 and 2016 and were transferred to the strain collection of the National Reference Laboratory (NRL) for Paratuberculosis, Friedrich-Loeffler-Institut (FLI) in Jena, Germany. The majority of the isolates were submitted from regional veterinary diagnostic laboratories of the German federal states, which obtained the isolates as part of routine diagnostics from fecal or tissue samples of different animal species. Others were directly isolated by the NRL from diagnostic samples or from samples obtained as part of field studies.

Most isolates came from cattle: 438 isolates from individual cattle in 243 herds, mainly dairy herds. Different numbers of cattle isolates (n = 1 to 28) per herd and herds of origin (n = 6 to 51) per federal state were available for genotyping (Table S1). In addition, 46 isolates from 44 individual animals of eight non-cattle host species and from one healthy human were characterized (Table S2). The origin of isolates was indicated by assignment to the numbered source herd (cattle isolates) or the numbered collection site/location (non-cattle isolates) within each federal state. The additional animal species included sheep (domestic sheep [*Ovis gmelini aries*], European mouflon [*Ovis gmelini musimon*], and Cameroon sheep [*Ovis aries aries*]), goat (*Capra aegagrus hircus*), red deer (*Cervus elaphus*), roe deer (*Capreolus capreolus*), fallow deer (*Dama dama*), Barbary sheep (*Ammontragus lervia*), European bison (*Bos bonasus*), and a miniature donkey (*Equus asinus f. asinus*), which was included as a non-ruminant species. The animals were free living (deer and mouflon) or held in domestic herds (sheep and goat), in zoological gardens, or were privately owned (sheep, goat, bison, and donkey). The only human isolate came from a healthy person. The year in which the individual isolates were collected is also listed in Tables S1 and S2.

Furthermore, 63 isolates (Table S3) originating from two to five tissues and/or fecal samples from 22 animals of 6 host species (cattle, bison, sheep, red deer, roe deer, and goat) were included to identify co-infections at the individual animal level (29 out of these 63 isolates were also included in the strain panels in Tables S1 and S2). Tissue samples originated from the intestinal tract (ileum, intestinal mucosa, jejunum, jejunal lymph node (LN), caecum, ileo-caecal LN, and mesenterial LN) or from extraintestinal tissues (liver, liver LN, spleen, muscle, udder, milk, or lung of fetus).

Some isolates, indicated with references in Tables S1–S3, were also included in previous studies [8,10,94,97,98].

### 4.2. Map Strain Isolation, Cultivation, and Subspecies Identification

#### 4.2.1. Strain Isolation, Cultivation, Sub-Cultivation

The *Map* strains were originally isolated from feces and different tissues of cattle and non-cattle, as well as from boot swabs and slurry collected in cattle barns of individual herds according to the standardized protocol described in the official manual of diagnostic procedures of the National Reference Laboratory (NRL) for Paratuberculosis published by the FLI [99], with some modifications. Briefly, feces and slurry (3 g) were decontaminated for 48 h using 30 mL 0.75% hexadecyl pyridinium chloride (HPC) solution (Sigma Aldrich, Taufkirchen, Germany), and crushed tissue (1 g) was decontaminated for 24 h using 7 mL 0.9% HPC, both at room temperature. After centrifugation (20 min, 1880× *g*) and discarding of the supernatant, the pellet was resuspended in 1 mL 1 × PBS (phosphate-buffered saline), and 0.2 mL of this solution was inoculated onto slants of Herrold`s egg yolk-medium (FLI, Jena, Germany) with ANV (Amphotericin, nalidixic acid, and Vancomycin), Löwenstein-Jensen *Map* medium, and Stonebrink *Map* medium with pyruvate (Artelt-Enclit, Borna, Germany), all supplemented with 2 mg/L of Mycobactin J and incubated at 37 °C for three to twelve months. The Mycobactin J dependence of these isolates was confirmed to exclude other mycobacterial species as sources. Characteristic colonies appeared after four to six weeks at the earliest. Boot swab samples (n = 17) were processed differently [100] in different laboratories. Fecal material was either rinsed off with sterile PBS and centrifuged prior to decontamination with 0.75% HPC solution, or about 5 g of cut boot swabs was placed directly in the 0.75% HPC solution and processed further, as described above [100].

After confirmation of *Map* subspecies, as described below, culture material for each *Map* isolate was cryo-conserved in Middlebrook bouillon (MB 7H9) supplemented with Mycobactin J (Allied Monitor, Inc. Fayette, MO, or IDvet, Grabels, France), glycerol, 10% Middlebrook Oleic acid, Albumin, Dextrose, and Catalase (OADC) enrichment (Becton Dickinson) and PANTA (Polymyxin B, Amphotericin B, nalidixic acid, Trimethoprim, Azlocillin—all SIGMA-Aldrich, Taufkirchen, Germany) at −80 °C or was used directly for genotyping.

*Map* isolates supplied by other German Federal State laboratories and cryo-conserved isolates were sub-cultivated at the FLI in Middlebrook bouillon (MB 7H9) supplemented with Mycobactin J and 10% Middlebrook Oleic acid, Albumin, Dextrose, and Catalase (OADC) enrichment (Becton Dickinson, Sparks, MD, USA), tested for their subspecies identity, and were mass-cultivated on solid media as mentioned above.

#### 4.2.2. DNA Extraction for PCR Reactions

DNA was isolated from several colonies of culture material after decontamination (20 min at 80 °C), followed by ultrasonication (35 kHz, 10 min), heating (10 min at 100 °C in a heating block), and centrifugation for 5 min at 12,000 rpm (15,300× *g*). The supernatant was centrifuged again for 5 min at 12,000 rpm, and DNA concentration was measured using a NanoDrop 1000 spectrophotometer (Thermo Fisher Scientific, Wilmington, DE, USA). For the IS*900*-RFLP analysis described below, DNA was isolated using the cetyltrimethylammonium bromide (CTAB) method (see Section 4.3.1).

#### 4.2.3. Molecular *Map* Identification

After 4 to 12 weeks (or longer for sheep and some cattle isolates), DNA was extracted from visible bacterial growth in cultures and characterized using PCR assays that speciate *Mycobacterium* (*M.*) *avium* complex (MAC) bacteria. These assays target the reliable subspecies-specific markers: IS*900* [101], IS*1245* [102], and IS*901* [103]. *Map* was detected (IS*900* positive, IS*1245* negative, and IS*901* negative) and distinguished from *Mah* (IS*900* negative, IS*1245* positive, and IS*901* negative) and *M. avium* subsp. *avium* (IS*900* negative, IS*1245* positive, and IS*901* positive).

### 4.3. Molecular Genotyping

*Map*-isolates were differentiated into C- or S-type and within the S-type group into subtype I or III by targeting the *gyrA* gene (*gyrA*-2 and *gyrA*-3) using two different PCR assays with subsequent sequencing [21]. These assignments were later confirmed by IS*900*-RFLP-profile analysis [58] when a large amount of high-quality DNA was available. Three complementary typing techniques were applied for more detailed sub-typing: IS*900*-RFLP, MIRU-VNTR, and SSR analysis.

#### 4.3.1. IS*900*-RFLP Analysis

IS*900*-Restriction Fragment Length Polymorphism (RFLP) analysis is a classical Southern blot method used for differentiation of *Map* strains [18] using the *Map*-specific IS*900* element, which is present 16 to 22 times in the genome [31]. DNA is cut into thousands of fragments by specific restriction enzymes, separated according to their sizes in an agarose gel, and hybridized with a *Map*-specific IS*900* probe. Following immunological detection and visualization, specific IS*900* band patterns are revealed for each isolate. The IS*900*-RFLP patterns differ between the C-type and S-type (subtypes I and III) lineage(s) and within the S-type lineage due to different genome structures and numbers of IS*900* elements. Within the C-type lineage, genomes show strong synteny and an identical or nearly identical number of IS elements [31,69]. Furthermore, it is assumed that the chromosomal localization of the IS*900* elements (IS*900* loci) within the main *Map*-lineages is conserved [104]. Therefore, different IS*900* patterns are presumably also based on differences (SNVs/SNPs and epigenetic modifications [105]) in the DNA of each isolate at the restriction sites of the respective digestion enzyme, leading to associated changes in the DNA fragment lengths in gel.

For IS*900*-RFLP, genomic DNA of high quality was required. It was extracted using a modification of the standardized CTAB method [106]. This modified method included treatment of killed bacteria resuspended in 500 µL of TE buffer (10 mM Tris-HCl, 10 mM EDTA, pH 8.0) with 1 µL of RNAse A (10 mg/mL) and 50 µL of lysozyme (10 mg/mL) and incubation overnight (rather than for 1 h) at 37 °C. In contrast to the standard CTAB method [106], the treatment with proteinase K and SDS was followed by the addition of 100 (rather than 80) µL of CTAB/NaCl solution and also 100 µL of 5 M NaCl solution. Three additional wash steps with 70% ethanol and centrifugation at 12,000 rpm (15,300× *g*) at 4 °C (four wash steps in total) were performed on the DNA pellets, including drying DNA for 20 min at 60 °C (rather than air drying), and dissolving the DNA in EB buffer (10 mM Tris-HCl, pH 8.5) (rather than 0.1X TE) for 1 h at 60 °C, which were also part of the modifications. DNA isolation for the other two typing methods was described above.

IS*900*-RFLP analysis was by Sothern blotting as previously described in [52]. Briefly, two micrograms of purified genomic DNA from each isolate was digested with *Bst*EII and *Pst*I (New England Biolabs, Frankfurt am Main, Germany). The resulting DNA fragments were separated by horizontal electrophoresis on a 240 mm 1% (*w/v*) agarose gel at 65 V for 16 h, vacuum blotted onto a HybondTM-N nylon membrane (Amersham Biosciences, Little Chalfont, UK), and hybridized with a digoxigenin (DIG)-labeled DNA probe specific for insertion element IS*900* (Southern blotting). The target molecules were subjected to immunological detection with anti-DIG antibodies conjugated to alkaline phosphatase (DIG luminescence detection kit; Roche Diagnostics, Mannheim, Germany). The CSPD detection system was used to visualize the hybridized probe (Roche Diagnostics, Mannheim, Germany). Hyperfilm ECL (GE Healthcare, Freiburg, Germany) was exposed after placement on the nylon membrane and developed for an optimal exposure time of 1 to 30 min.

The probe was prepared by PCR amplification using the forward primer 5′-TGG ACA ATG ACG GTT ACG GAG GTG G-3′ and the reverse primer 5′-GAT CGG AAC GTC GGC TGG TCA GGA T-3′ recommended in the standardized IS*900*-RFLP protocol [58] with genomic DNA of *Map* strain ATCC 19698 as the source. PCR was conducted with a digoxigenin (DIG)-labeled deoxynucleoside triphosphate (dNTP) mixture (DIG DNA labeling mix; Roche Diagnostics) [52]. The amplification product was purified with a QIAquick gel extraction kit (Qiagen, Hilden, Germany). This probe was a specific IS*900* fragment of 453 bp containing no *Pst*I and no *Bst*EII digestion site.

The resulting IS*900*-RFLP genotypes consisted of two robust band patterns. Known patterns were designated with the nomenclature described previously after *BstE*II digestion with “C” [cattle type] or with “I” [intermediate type (=subtype III)] and after *Pst*I digestion with P, together with sequential numbers [8,10,19,29,51,52,58,59,107,108,109]. To simplify the nomenclature, the former IS*900*-RFLP (*Bst*EII) patterns Cn1-Cn9 [8,29,52] were changed to C41-C49. New patterns were given new designations.

#### 4.3.2. MIRU-VNTR Typing

Mycobacterial interspersed repetitive-unit–variable-number tandem repeat (MIRU-VNTR) typing is based on the *Map* strain depending on variation in the number of different short tandem repeat structures comprising repetitive nucleotide sequences (minisatellites of 18–58 nt) at multiple conserved (putative intergenic) loci throughout the *Map* genome [34,50,110].

MIRU-VNTR typing in this study was performed using specific PCR reactions targeting eight mini-satellite regions (loci 292, X3, 25, 47, 3, 7, 10, and 32), [50]. The PCR conditions and annealing temperatures were updated [111]. The number of copies of mycobacterial interspersed repetitive units (MIRUs) at loci 292 and X3 and of variable number tandem repeats (VNTRs) at loci 25, 47, 3, 7, 10, and 32 were determined according to the sizes of the amplicons after separation by electrophoresis using 1.5% agarose gels (universal agarose, Bio&Sell, Feucht, Germany). Different-sized alleles were classified using an allele-calling table [112,113]. The numbers of these repeats were arranged according to the mentioned order of loci and assigned to given numerical codes according to the INRA Nouzilly MIRU-VNTR (INMV) nomenclature for MIRU-VNTR patterns, the so-called INMV profiles, available in the freely accessible MAC-INMV-SSR database http://mac-inmv.tours.inra.fr (accessed on 1 September 2024) [78]. For new numerical codes, new INMV profile numbers were assigned by the database managers upon request. Additionally, to identify polymorphisms at VNTR locus 7 for strains with unusual amplicon sizes, PCR products were purified using the QIAquick PCR purification kit (Qiagen, Hilden, Germany) according to the manufacturer’s instructions and sequenced at Eurofins Genomics GmbH (Konstanz, Germany). Resulting sequence chromatograms were compared with reference sequences [82]. Furthermore, to analyze unusual or unclear results at other VNTR loci, amplicons of PCRs with these regions were also sequenced. In our analysis, 8.6 tandem repeats (TR) at VNTR32 locus were interpreted as 8 TR.

#### 4.3.3. SSR Analysis

Short-sequence repeat (SSR) analysis of *Map* is based on microsatellite structures with strain-depending variations in the numbers of mono-, di-, and trinucleotide repeats at specific loci in the genome [46]. Depending on the number and stability of the selected polymorphic SSR loci analyzed, the method is very useful for distinguishing *Map* isolates, as well as in combination with other typing methods [39,40,85]. The impact of the copy number of the SSR at the different genome loci on the expression of neighboring genes is not yet known [46].

SSR analysis was applied according to the published procedure [8,46]. The microsatellite structures at SSR locus 1 (G1 repeats), locus 8 (GGT repeats), and locus 9 (TGC repeats) were chosen because these are the most informative SSR loci [39,40,46,85]. The same primers were used as published [46]. For amplification of G1 repeats, a specific polymerase (Herculase II Fusion DNA polymerase; Agilent Technologies, Cedar Creek, TX, USA) was chosen to minimize the risk of polymerase slippage at poly(G) motifs. PCR products were purified using the QIAquick PCR purification kit (Qiagen, Hilden, Germany) according to the manufacturer’s instructions and sequenced at Eurofins Genomics GmbH (Konstanz, Germany).

The number of specific repeats at these loci was given a numerical code. Isolates with 11 or more G1 repeats at locus 1 were designated as ≥11 because of slipping effects of the polymerase described previously [39,72]. Locus 2 was not included, as it was found to be unreliable in previous studies [8,72]. The three SSR loci used for this work are referred to below as SSR1, SSR8, and SSR9.

#### 4.3.4. Analysis of Typing Results and Discriminatory Power

Altogether, this complementary genotyping method used 13 different target structures within the *Map* genome and resulted in two band patterns of IS*900*-RFLP, repeat numbers at eight specific MIRU-VNTR loci, and repeat numbers at three specific SSR loci. Isolates that differed in at least one or more of these targets/genomic markers were considered to have distinct genotypes.

The discriminatory power of genotyping (IS*900*-RFLP, MIRU-VNTR, and SSR typing alone and combined) was calculated using the discriminatory index (DI) according to Hunter and Gaston [114], as described before [52]. The DI indicates the probability of each typing approach to discriminate between two epidemiologically unlinked strains of the German *Map* population in this study. Only epidemiologically unrelated (=independent) strains were included in these calculations. This means that if a genotype occurred in more than one isolate in the same herd, it was only considered once. Therefore, depending on the typing method (separate or combined), the number of independent strains for calculation varied. Isolates with a mixed genotype were included as two individual genotypes.

### 4.4. Comparison of Strains and Analysis of Phylogeny

Phylogenies were constructed using the unweighted-pair group method with arithmetic averages (UPGMA) to compare categorical values for the combined genotypes. The relationships of all identified genotypes of strains, their distribution in cattle herds and other hosts or federal states, and the pairwise relationships of strains within federal states for epidemiological investigations were visualized with similarity matrice-based dendrograms and minimum spanning trees (MST). The dendrograms and MSTs were generated using BioNumerics software version 7.5 created by Applied Maths NV (St-Martens-Latem, Belgium).

## 5. Conclusions

This study of isolates spanning 24 years gave an overview of the diversity of 507 *Map* isolates belonging to the C-type group, and, in addition, 11 isolates of the S-type group originating from a country in the middle of Europe where paratuberculosis is endemic in dairy cattle and has a variable prevalence and long history. This study was based on a very large *Map* strain collection, started with isolates from 1993. The high discriminatory power of the combined multi-locus genotyping, consisting of IS*900*-RFLP (*Bst*EII, *Pst*I), MIRU-VNTR, and SSR, enabled the detection of high *Map* diversity in Germany. *Map* transmission between cattle herds within or between federal states and between different hosts, including domestic and wildlife animals, was suggested. There were predominant genotypes distributed in cattle herds in most of the federal states that also had infected non-cattle species. Two or more genotypes revealed in isolates from individual animals, single herds, and locations indicated possible polyclonal infections. Similar genotypes with differences at only one typing target in isolates from one origin (single isolates, animals, and herds) suggested divergent evolution. There was no significant association of genotypes with their geographical origin. This study could not match inferences about *Map* transmission facilitated by WGS-based studies, but the modularity of the method used (RFLP IS*900*, MIRU-VNTR, and SSR) allowed for comparisons with typing using one or more of these methods carried out by other groups, facilitating comparisons of genotype distributions within and across national boundaries. In comparison to the results of high-resolution WGS-based studies, it would be interesting to find out what role the polymorphisms at the MIRU-VNTR and SSR loci and the SNPs or variable genome structure responsible for the different RFLP patterns play in revealing *Map* transmission and phylogenetic relationships. Further studies will compare the current combined genotyping results with those of whole-genome core SNP-based analysis of a selection of these *Map* strains to clarify the validity/importance of the observed clustering of combined genotypes.

## Figures and Tables

**Table 1:** Combined genotypes (GTs) detected in German *Mycobacterium* (*M.*) *avium* subsp. *paratuberculosis* strains isolated from different host species between 1993 and 2016.

GT	RFLP ^a^	VNTR ^b^	INMV ^c^	SSR ^d^	[n]	Host
1	C1-P1	42332228	1	7-4-4	17	C, G
2	C1-P1	42332228	1	7-4-5	16	C, S, D
3	C1-P1	42332228	1	8-4-5	1	G
4	C1-P1	32332228	2	7-4-4	53	C, G, S
5	C1-P1	32332228	2	7-4-5	13	C, S
6	C1-P1	32332228	2	7-5-5	1	C
7	C1-P1	32332228	2	9-5-5	2	C
8	C1-P1	32332228	2	10-5-5	1	C
9	C1-P1	32332228	2	≥11-5-5	7	C
10	C1-P1	32332228	2	≥11-5-6	1	C
11	C1-P1	32332218	3	7-4-4	3	C
12	C1-P1	32332229	4	7-4-4	1	C
13	C1-P1	42332218	5	7-4-4	1	C
14	C1-P1	42332218	5	7-4-5	1	C
15	C1-P1	32332128	6	7-4-5	1	H
16	C1-P1	32332128	6	≥11-4-5	9	C
17	C1-P1	32332(5) ^e^ 28	16 *	7-4-4	2	C
18	C1-P1	22522228	12	7-4-4	10	C
19	C1-P1	22332228	13	7-4-4	2	C
20	C1-P1	42332128	19	7-4-4	3	C
21	C1-P1	32522(3) ^f^ 28	30 *	7-4-4	1	C
22	C1-P1	32522228	33	7-4-4	29	C, G, S
23	C1-P1	22522226	80	7-4-4	4	C
24	C1-P1	32322228	117	7-4-4	3	C
25	C1-P1	12322226	132	7-4-4	1	C
26	C1-P1	12522226	133	7-4-4	1	C
27	C1-P1	12322126	134	7-4-4	1	C
28	C1-P1	22522128	136	7-4-4	4	C
29	C1-P1	52332228	139	9-4-5	1	DO
30	C1-P1	42322228	141	7-4-4	1	C
31	C1-P1	32422128	142	7-4-4	1	C
32	C1-P1	22522218	217	7-4-4	1	C
33	C1-P1	22322226	262	7-4-4	3	C, G
34	C1-P1	42332226	263	7-4-5	1	C
35	C1-P2	32332228	2	7-4-3	2	C
36	C1-P2	22332228	13	7-4-3	1	C
37	C1-P3	42332128	19	7-4-4	23	C, G, D
38	C1-P3	42332128	19	8-4-4	1	C
39	C1-P3	43332128	143	7-4-4	8	C
40	C1-P3	43332118	264	7-4-4	1	C
41	C1-P7	32332228	2	7-5-5	1	D
42	C1-P10	42332128	19	7-4-4	1	C
43	C2-P18	32522228	33	7-4-4	2	C
44	C5-P1	32332228	2	≥11-5-5	1	C
45	C5-P7	32332228	2	7-5-5	1	D
46	C5-P8	42332228	1	7-4-4	1	C
47	C9-P2	42332228	1	7-4-4	1	C
48	C9-P2	32332228	2	7-4-4	1	C
49	C10-P6	42332228	1	7-4-4	1	C
50	C10-P6	42332218	5	7-4-4	2	C
51	C10-P6	42322228	141	7-4-4	1	C
52	C10-P17	42332218	5	7-4-4	1	C
53	C12-P3	42332128	19	7-4-4	1	C
54	C13-P18	32332227	191	7-4-4	1	C
55	C16-P12	42332128	19	7-4-4	1	C
56	C17-P8	42332228	1	7-4-4	12	C
57	C17-P8	32332228	2	7-4-4	3	C, D
58	C17-P8	42332226	263	7-4-4	1	C
59	C17-P8	42342228	265	7-4-4	1	C
60	C17-P9	42332228	1	7-4-4	1	C
61	C17-P9	42332128	19	7-4-4	8	C, S
62	C17-P9	12332228	248	7-4-4	1	C
63	C18-P1	32522228	33	7-4-4	1	C
64	C18-P16	42332228	1	7-4-5	7	C
65	C18-P16	32332228	2	6-4-4	2	C, D
66	C18-P16	32332228	2	7-4-4	1	C
67	C18-P16	32332228	2	10-5-5	2	C
68	C18-P16	42332218	5	7-4-4	1	C
69	C18-P16	22332228	13	≥11-5-5	1	C
70	C18-P16	22522226	80	7-4-4	2	C
71	C22-P1	32332228	2	7-4-4	1	D
72	C22-P11	42332128	19	7-4-4	1	C
73	C22-P20	22522228	12	7-4-4	1	C
74	C24-P19	32522228	33	7-4-4	4	C
75	C27-P1	42332228	1	7-4-5	2	C
76	C27-P3	42332128	19	7-4-4	1	C
77	C29-P3	42332128	19	7-4-4	1	C
78	C41-P1	32332228	2	7-5-5	2	C
79	C41-P1	32332128	6	7-5-5	1	C
80	C41-P7	32332228	2	7-5-5	14	C, D, S, B
81	C41-P7	32332128	6	7-5-5	1	C
82	C42-P3	42332128	19	7-4-4	1	C
83	C43-P3	42332128	19	7-4-4	3	C
84	C44-P1	32332228	2	7-4-4	1	C
85	C44-P1	32522228	33	7-4-4	1	C
86	C45-P1	32332228	2	≥11-5-5	1	C
87	C46-P1	42332229	190	8-4-4	4	C
88	C47-P1	42332228	1	7-4-4	1	C
89	C48-P18	32522228	33	7-4-4	1	C
90	C49-P17	42332218	5	7-4-4	1	C
91	I2-P14	22331(1.5) ^g^ 18	220	7-3-4	1	S
92	I6-P13	42131(1.5)18	266	7-3-4	4	S, C
93	I7-P14	52331(1.5)18	267	7-3-3	1	S

^a^ IS*900*-RFLP patterns after digestion with *Bst*EII and *Pst*I. Individual RFLP patterns are shown in Figures S1 and S2. ^b^ Numerical code, including the number of tandem repeats at MIRU-VNTR loci 292, X3, 25, 47, 3, 7, 10, and 32. ^c^ INMV profiles designation. ^d^ Number of short-sequence repeats (SSR) at [G] locus 1, [GGT] locus 8, and [TGC] locus 9. ^e^ In addition to three repeats at MIRU-VNTR locus 7, between repeat 2 and 3, a second back flanking region of 34 nucleotides (nt) was found by sequencing, resulting in amplicon size appearing like five repeats. ^f^ In contrast to three true repeats of locus 7, the 3rd repeat consisted of only of 12 nt out of 22 nt of the specific sequence, along with 12 nt of the back-flanking sequence region between the 2nd and 3rd repeat, resulting in an amplicon size appearing like three repeats. INMV16* and INMV30*—profile assignments were based on amplicon sizes at all 8 loci. Due to the altered repeat sequences at locus 7 as explained above, the profiles were marked with *. ^g^ At MIRU-VNTR locus 7 in addition to one repeat 12 nt were identified by sequencing, appearing like 1.5 repeats in the gel. Underlined—the five predominant genotypes in German cattle. [n]—Number of epidemiologically unrelated isolates; C—cattle, G—goat, S—sheep, D—deer, H—human, DO—donkey, B—bison.

**Table 2:** New numerical codes determined by MIRU-VNTR typing of German isolates.

INMV Code	INMV Profile	Map-Type	Host Origin	Herd, Location (Loc) ^a^
22322226	262	C	cattle, goat	BB-2, RP-29, BB/Loc3
42332226	263	C	cattle	RP-33, RP-34
43332118	264	C	cattle	SL-6
42342228	265	C	red deer	NRW/Loc13
42131(1.5) ^b^ 18	266	S	sheep, cattle	NRW/Loc11, TH/Loc24; BY-2, BY-5
52331(1.5) ^b^ 18	267	S	sheep	NRW/Loc10
325223*28	30*	C	cattle	TH21

^a^ Cattle herds are numbered per federal state, and the sources of the other species are numbered by locations. For more details, see Figure 1 and Tables S1 and S2. ^b^ At VNTR7, in addition to one repeat, an imperfect read with 12 nucleotides (CGT TCG GCG CGC) was identified by sequencing, first mentioned for S-type strains in Germany in 2009 [10]. 3* At VNTR7, in addition to two repeats, a 24 bp region was identified by sequencing, different from original repeat sequence. On agarose gel, the PCR product appeared like 3 repeats. 30* The respective code (325223*28) with specific sequence at VNTR7 was designated as type INMV30*.

**Table 3:** Predominant *M. avium* subsp. *paratuberculosis* genotypes in cattle herds.

Genotype	IS*900*-RFLP/MIRU-VNTR/SSR	Herds * [n]	Federal State [n]
GT4	C1-P1/INMV2 (32332228)/7-4-4	48 (20%)	9
GT22	C1-P1/INMV33 (32522228)/7-4-4	26 (11%)	10
GT37	C1-P3/INMV19 (42332128)/7-4-4	21 (9%)	8
GT1	C1-P1/INMV1 (42332228)/7-4-4	16 (7%)	7
GT2	C1-P1/INMV1 (42332228)/7-4-5	14 (6%)	7

* In total, 119 of the 243 tested herds (49%) harbored at least one of these genotypes. A total of 7 out of 243 herds (3%) had dual infections with two of these predominant genotypes identified.

**Table 4:** Mixed genotypes in cattle isolates.

No.	INMV Code	INMV Profile ^a^	SSR	IS*900*-RFLP ^b^	GT ^c^	Isolate (n)	Herd ^d^ (n)
1	(3+4)2332228	2 + 1	7-4/5-4/5	C1-(P1 + *P3*)	-	1	NRW-12
2	(3+4)2332(1+2)28	2 + 19	7-4-3/4	C1-(P2 + P3)	35 + 37	1	RP-23
3	42332(1+2)28	1 + 19	7-4-4/5	C1-(P1 + *P3*)	2 + 37	1	RP-31
4	4233222(6+8)	263 + 1	7-4-5	C1-P1	34 + 2	1	RP-33
5	4233222(6+8)	263 + 1	7-4-4	C17-P8	58 + 56	2	RP-34
6	32(3+5)22228	117 + 33	7-4-4	C1-P1	24 + 22	1	SL-2
7	433321(1+2)8	264 + 143	7-4-4	C1-P3	40 + 39	1	SL-6
8	32332(5*+2)28	16 * + 2	7-4-4	C1-P1	17 + 4	2	TH-42,43

^a^ INMV profiles were assumed based on other identified profiles in herds of origin. ^b^ IS*900*-RFLP patterns after digestion with *Bst*EII and *Pst*I; ^c^ suspected mixed genotypes; ^d^ herd designation—see Table S1; *P3*—for the strains with the mixed INMV profiles (2 + 1) and (1 + 19), the *Pst*I pattern P3 was identified, which may also contain the profile P1, since it only has one additional band and is otherwise identical. * An altered repeat sequence was detected on VNTR locus 7, but on the agarose gel it appeared as five repeats. The profile 323325*28 was therefore designated as INMV16* (see also Table 1).

**Table 5:** Different combined genotypes (GT) in various tissues and feces of nine single animals.

N° ^a^	Host	Origin ^a^	Isolate	Tissue Origin	RFLP ^b^	INMV ^c^	Code ^d^	SSR ^e^	GT
1	Cattle	TH-1	01A0432	Jejunal LN	C17-P9	19	42332128	7-4-4	61
			01A0435	Liver	C17-P9	19	42332128	7-4-4	61
			01A0441	Muscle	C1-P1	1	42332228	7-4-4	1
2	Cattle	TH-1	01A0534	Jejunal LN ^f^	C1-P1	2	32332228	7-4-5	5
			01A0535	Jejunal LN ^g^	C17-P9	19	42332128	7-4-4	61
3	Cattle	TH-1	02A0112	Feces	C17-P9	19	42332128	7-4-4	61
			02A0116	Ileo-caecal LN	C17-P9	19	42332128	7-4-5	-
			02A0117	Jejunal LN	C17-P9	19	42332128	7-4-4	61
			02A0118	Liver	C17-P9	19	42332128	7-4-4	61
			02A0119	Liver LN	C17-P9	19	42332128	7-4-4	61
4	Cattle	TH-1	02A1191	Feces	C17-P9	19	42332128	7-4-4	61
			02A1192	Ileum	C17-P9	19	42332128	7-4-4	61
			02A1194	Caecum	C16-P12	19	42332128	7-4-4	55
5	Cattle	TH-3	05A2326	Feces	C9-P2	1	42332228	7-4-4	47
			05A2327	Ileum LN	C9-P2	1	42332228	7-4-4	47
			05A2328	Spleen LN	C9-P2	2	32332228	7-4-4	48
			05A2330	Lung of foetus	C9-P2	1	42332228	7-4-4	47
6	Cattle	NRW-3	06A1283	Feces 1 *	C17-P8	2	32332228	7-4-4	57
			06A1284	Feces 2 *	C41-P7	2	32332228	7-5-5	80
7	Cattle	NRW-13	06A1281	Feces 1 *	C17-P9	19	42332128	7-4-4	61
			06A1282	Feces 2 *	C17-P8	1	42332228	7-4-4	56
8	Roe deer	NRW-Loc17	06A1278	Feces 2 *	C5-P7	2	32332228	7-5-5	45
			06A1279	Feces 3 *	C1-P7	2	32332228	7-5-5	41
9	Sheep	TH-Loc24	11MA0986	Mesenteric LN	I6-P13	266	42131(1.5)18	7-3-4	92
			11MA0987a	Feces 1 (LJ)	I6-P13	266	42131(1.5)18	7-3-4	92
			11MA0987b	Feces 2 (Her)	n.d.	2	32332228	7-5-5	-

^a^ N° of herd or location (Loc), as indicated in Tables S1 and S2; NRW—North Rhine-Westphalia, TH—Thuringia; ^b^ IS*900*-RFLP pattern after digestion with *Bst*EII and *Pst*I. ^c^ INMV—INRA Nouzilly MIRU-VNTR nomenclature for MIRU-VNTR profiles; ^d^ number of tandem repeats (TR) at MIRU-VNTR loci 292, X3, 25, 47, 3, 7, 10, and 32; ^e^ number of short-sequence repeats at loci 1[G]-8[GGT]-9[TGC]; ^f^ from visible lesions; ^g^ without visible lesions; LN—lymph node; * feces 1, 2, or 3—isolates originated from the same animal and sampling time, but were cultivated on different media; (LJ)—cultivated in Löwenstein–Jensen medium; (Her)—cultivated in Herrolds Egg Yolk Medium; n.d.—not detected; underlined—isolated from the same animal with different GT.

## Data Availability

The data generated and analyzed during this study are included in the Supplementary Material. Additionally, data are available from the corresponding author upon reasonable request.

## Supplementary Materials

The following supporting information can be downloaded at: https://www.mdpi.com/article/10.3390/ijms26115273/s1.

## Glossary

Isolate = the culture of multiple colonies resulting from decontamination, inoculation, and incubation of one specimen of distinct origin on media; genotype = a unique pattern differing at least 1 of 13 different target regions from other known types, as determined by the combined IS*900*-RFLP, MIRU-VNTR, and SSR genotyping assay used in this study; strain = a specific genotype identified one or more times in one or more herds or locations; independent strain = a specific genotype identified one or more times in one herd or source.
