# Supplementary material for: High Molecular Diversity of Mycobacterium avium subsp. paratuberculosis in Germany Revealed by Multitarget Genotyping
Source: Int J Mol Sci. 2025 May 30;26(11):5273. doi: 10.3390/ijms26115273 (PMC12155428; doi:10.3390/ijms26115273)
Supplement: Supplementary file 1 [file ijms-26-05273-s001.zip › ijms-3584899-supplementary.pdf]

Supplemented Material

Figure S1

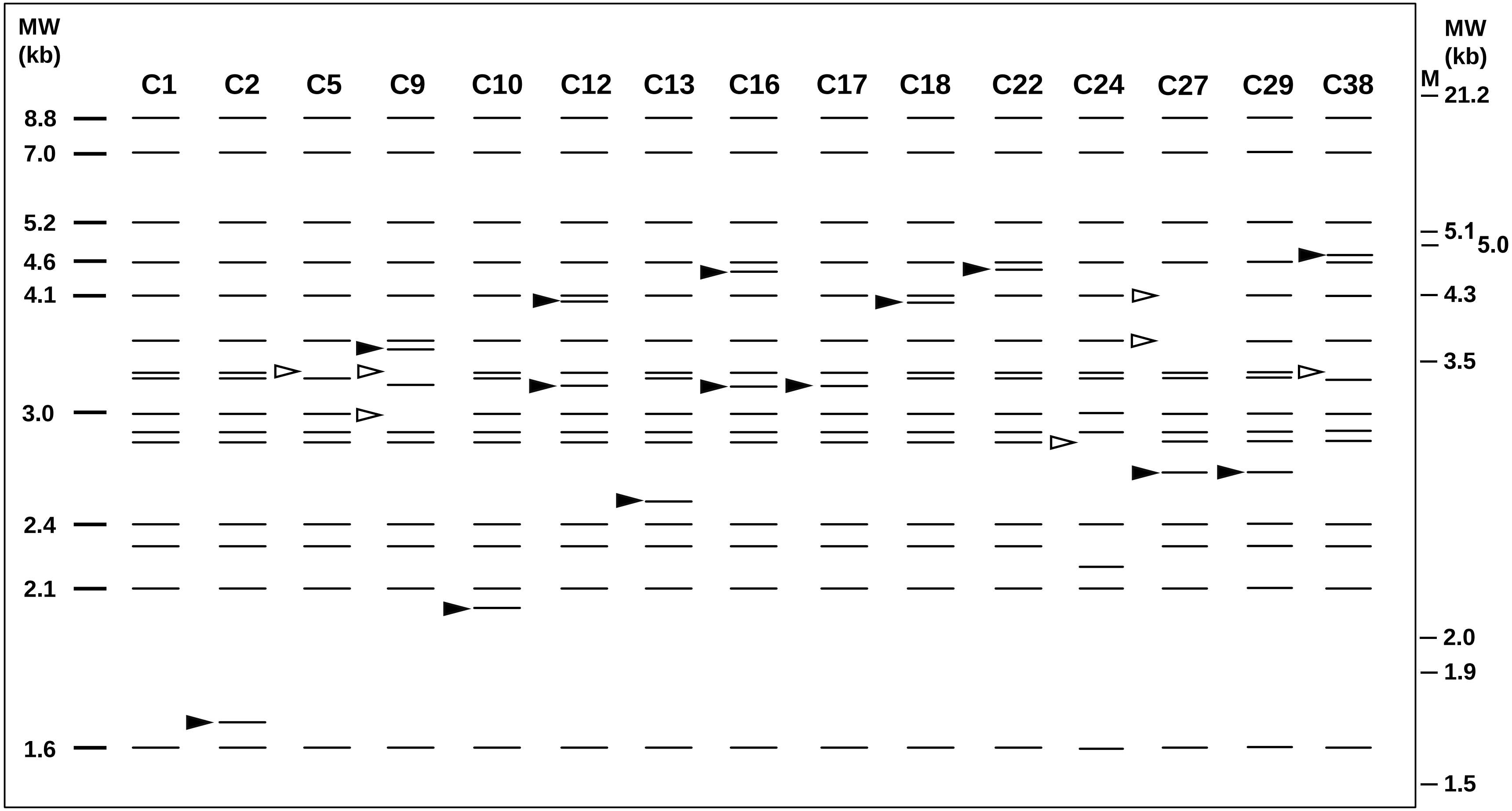

(1a)

Figure S1

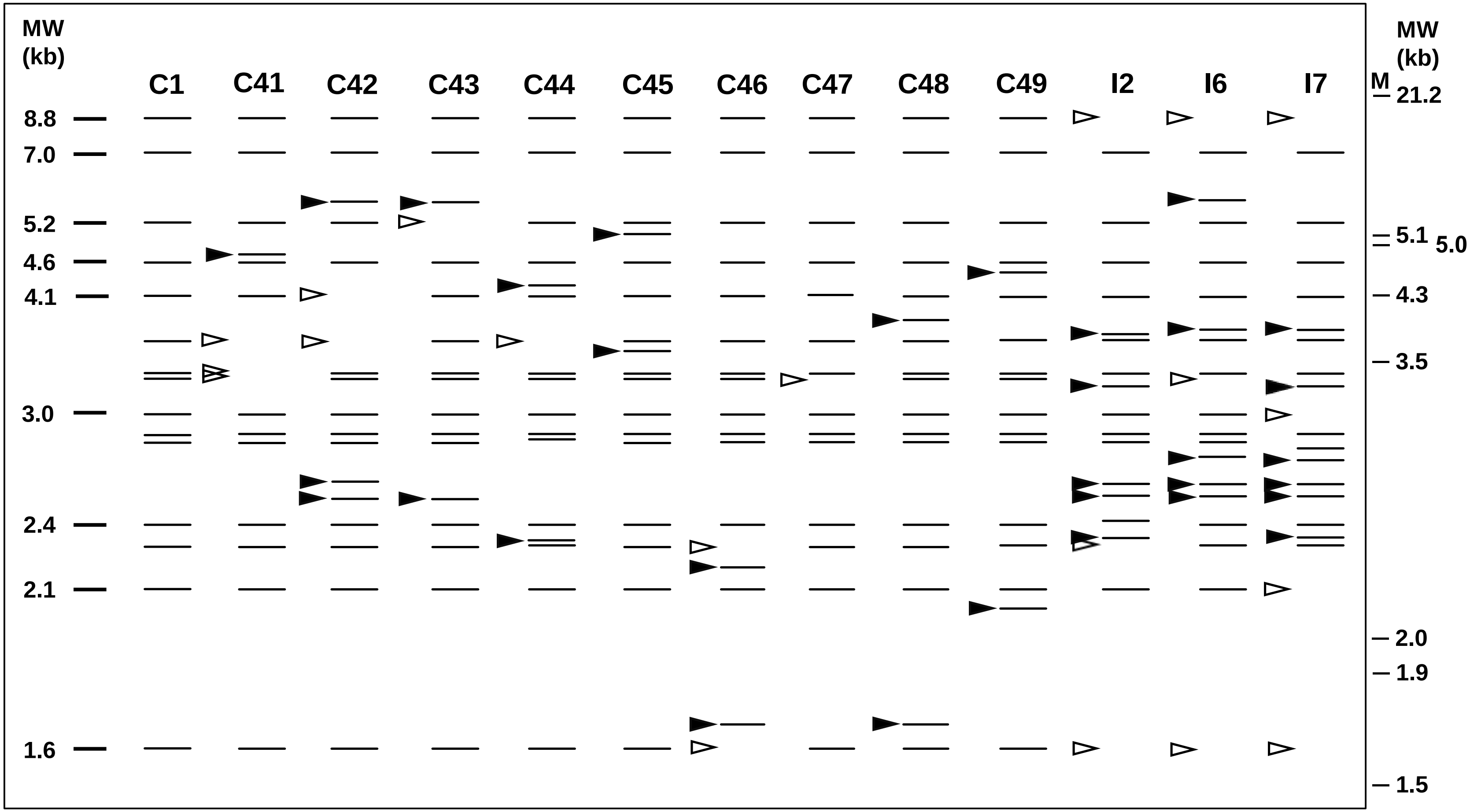
MW
(kb)
C1
C41
C42
C43
C44
C45
C46
C47
C48
C49
I2
I6
I7
M
MW
(kb)
8.8
7.0
5.2
4.6
4.1
3.0
2.4
2.1
1.6
21.2
5.1
5.0
4.3
3.5
2.0
1.9
1.5

(1b)

Figure S1. Schematic presentation of IS*900*-RFLP profiles of *Mycobacterium avium* subsp. *paratuberculosis* isolates from Germany after chromosomal DNA digestion with *Bst*EII. A digoxigenin-labelled specific 453 bp fragment of IS*900* without digestion site for *Bst*EII or *Pst*I was used as probe for the Southern blots. The arrowheads in the diagrams show the differences between profile C1 and the other types, (►) additional or shifted bands or (▷) the absence of bands. Lane M on the right represents the molecular weight marker III (Roche Diagnostics). Numbers on the left indicate sizes of the reference bands. Profiles were designated for C-type / subtype II isolates using "C" and S-type / subtype III isolates using "I".

(1a) Profiles of C-types are designated according to Pavlik et al., 1999, [58]. Many of these profile designations correspond with profiles/band patterns published by Overduin et al., 2004, [109], and Whittington et al., 2011, [19]: C1 = R01, C5 = R10 = CU7, C9 = R03, C10 = R04, C12 = R05 = CU4, C16 = R34, C17 = R09, C18 = R13, C24 = CU5. Profile C38 was determined for DSM 44133 (ATCC 19698).

(1b) Profiles of C-types C41 –C43 correspond to Cn1 - Cn3 in Fritsch et al., 2008, [8]; C44, C46 - C49 correspond to Cn4, Cn6 - Cn9 in Möbius et al., 2017, [29]; profile C45 is new. Profile I2 was published first by De Lisle et al., 1993, [107] for ovine strain P467; profiles I6 and I7 were published before in Möbius et al., 2009, [10].

Figure S2

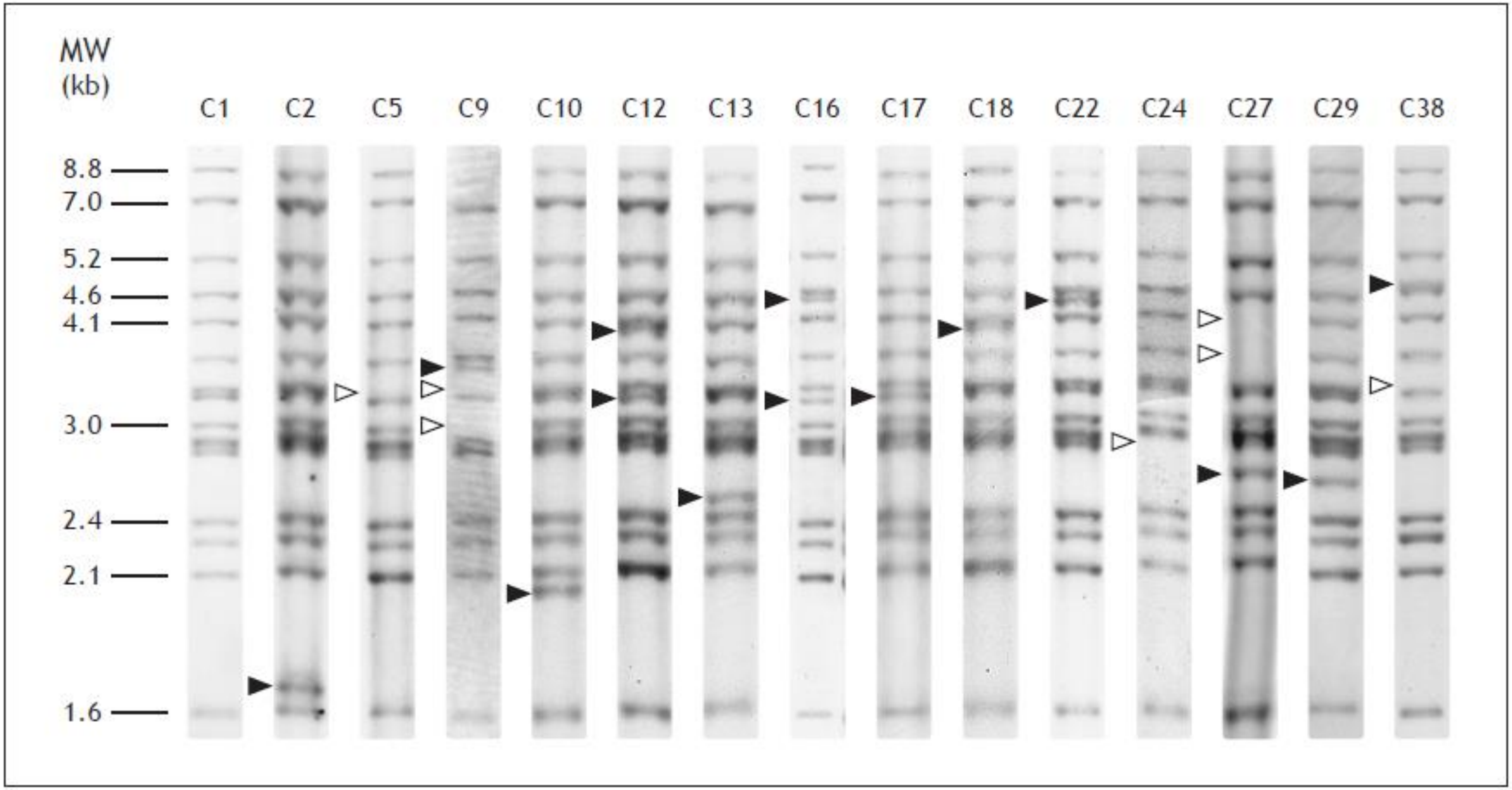

MW (kb)
C1
C2
C5
C9
C10
C12
C13
C16
C17
C18
C22
C24
C27
C29
C38
8.8
7.0
5.2
4.6
4.1
3.0
2.4
2.1
1.6

(2a)

Figure S2

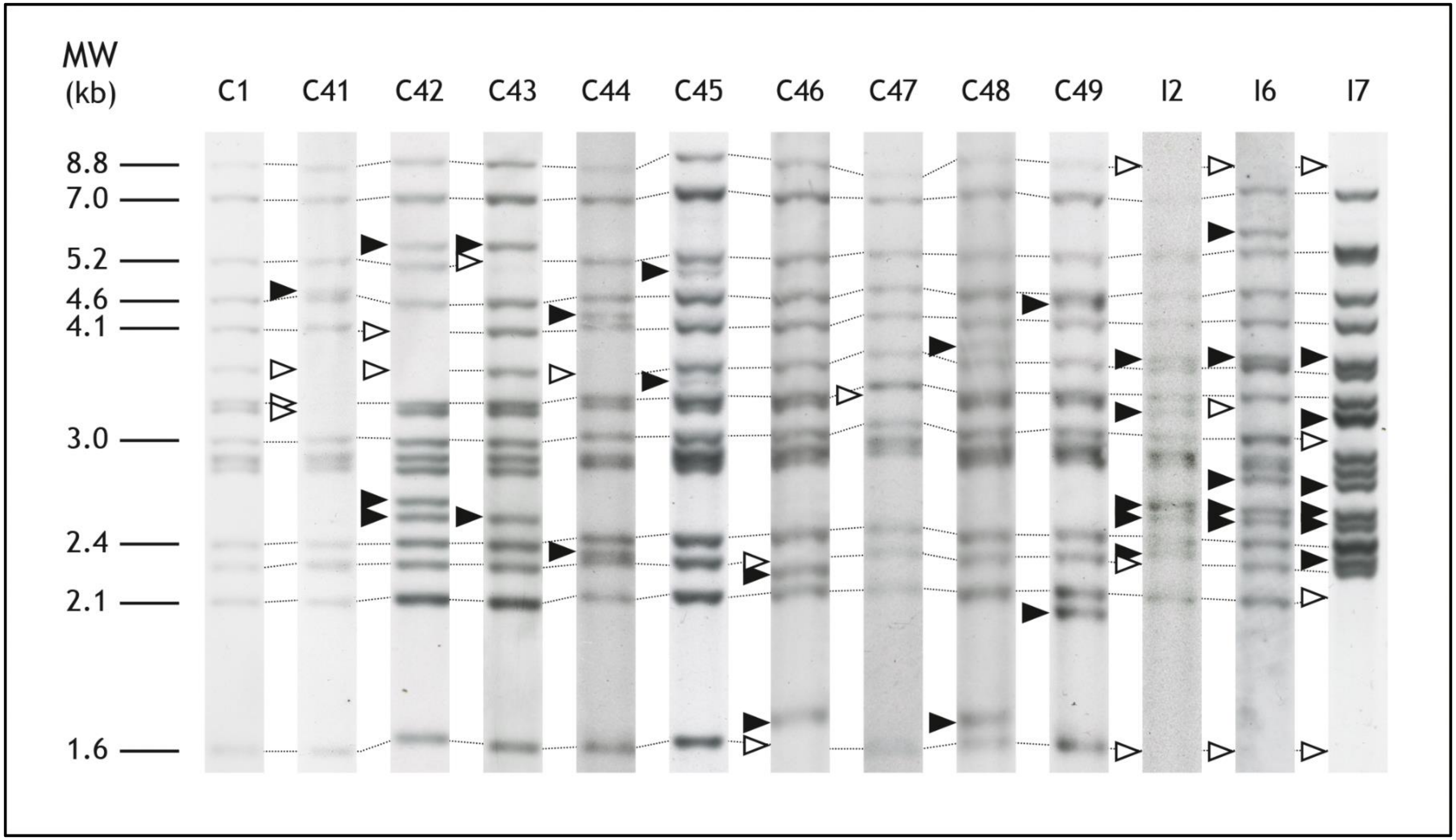

MW (kb)
C1
C41
C42
C43
C44
C45
C46
C47
C48
C49
I2
I6
I7
8.8
7.0
5.2
4.6
4.1
3.0
2.4
2.1
1.6

(2b)

Figure S2. Overview of IS*900*-RFLP profiles of *Mycobacterium avium* subsp. *paratuberculosis* isolates from Germany after chromosomal DNA digestion with *Bst*EII shown as images from different gels. A digoxigenin-labelled specific 453 bp fragment of IS*900* without digestion site for *Bst*EII or *Pst*I was used as probe for the Southern blots. The arrowheads in the images label the differences between profile C1 and the other types, (►) additional or shifted bands or (▷) the absence of bands. Numbers on the left indicate sizes of the reference bands. Profiles were designated for C-type / subtype II isolates using "C" and S-type / subtype III isolates using "I" and according to the references listed in legend of Figure S1. **(2a)** Images of IS*900*-RFLP (*Bst*EII) profiles C1, C2, C5, C9, C10, C12, C13, C16 - 18, C22, C24, C27, C29, C38. **(2b)** Images of IS*900*-RFLP (*Bst*EII) profiles C1, C41-49, I2, I6 and I7.

Figure S3

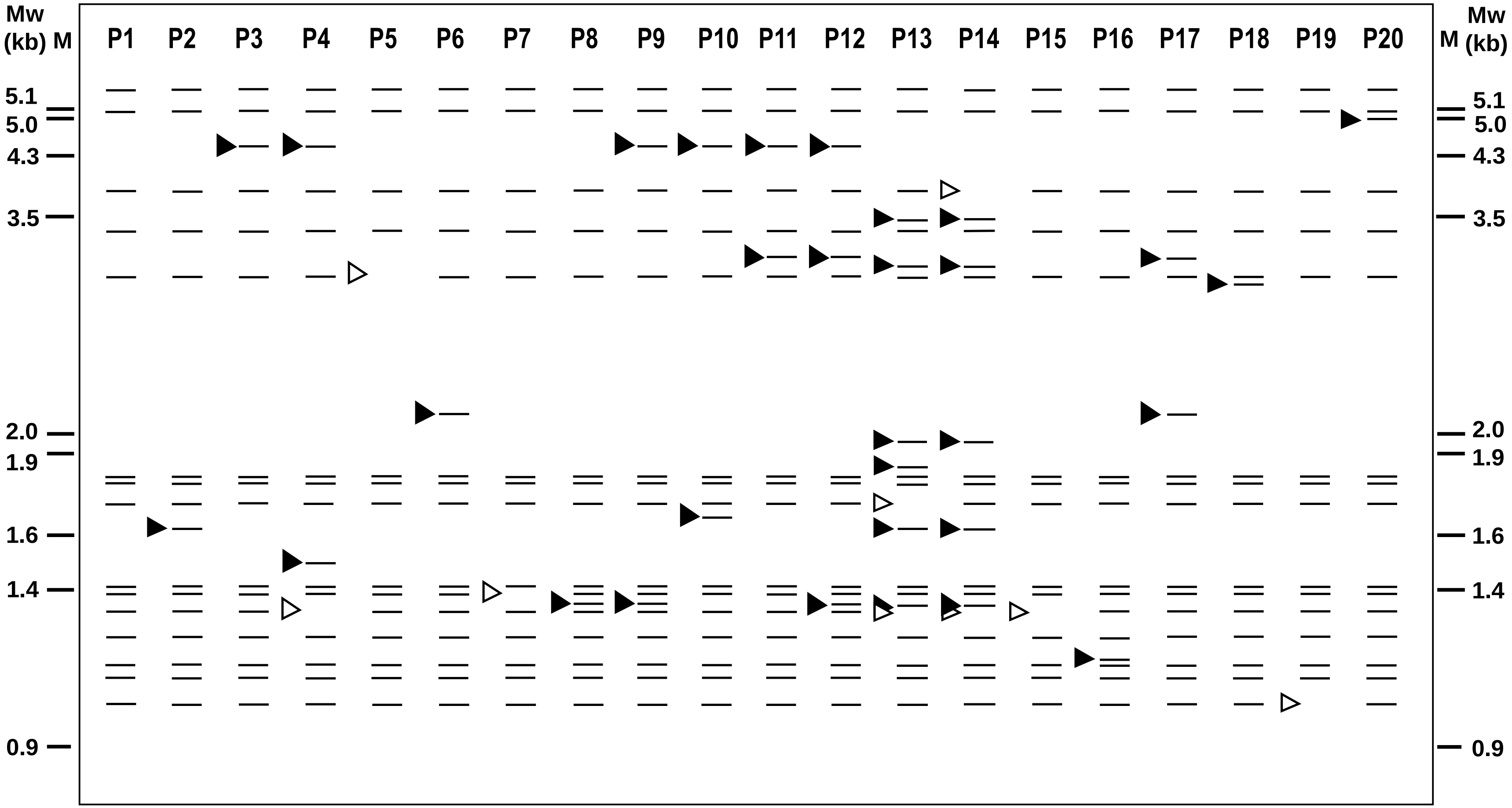

Mw (kb)
M
P1
P2
P3
P4
P5
P6
P7
P8
P9
P10
P11
P12
P13
P14
P15
P16
P17
P18
P19
P20
M
Mw (kb)
5.1
5.0
4.3
3.5
2.0
1.9
1.6
1.4
0.9

(3a)

Figure S3

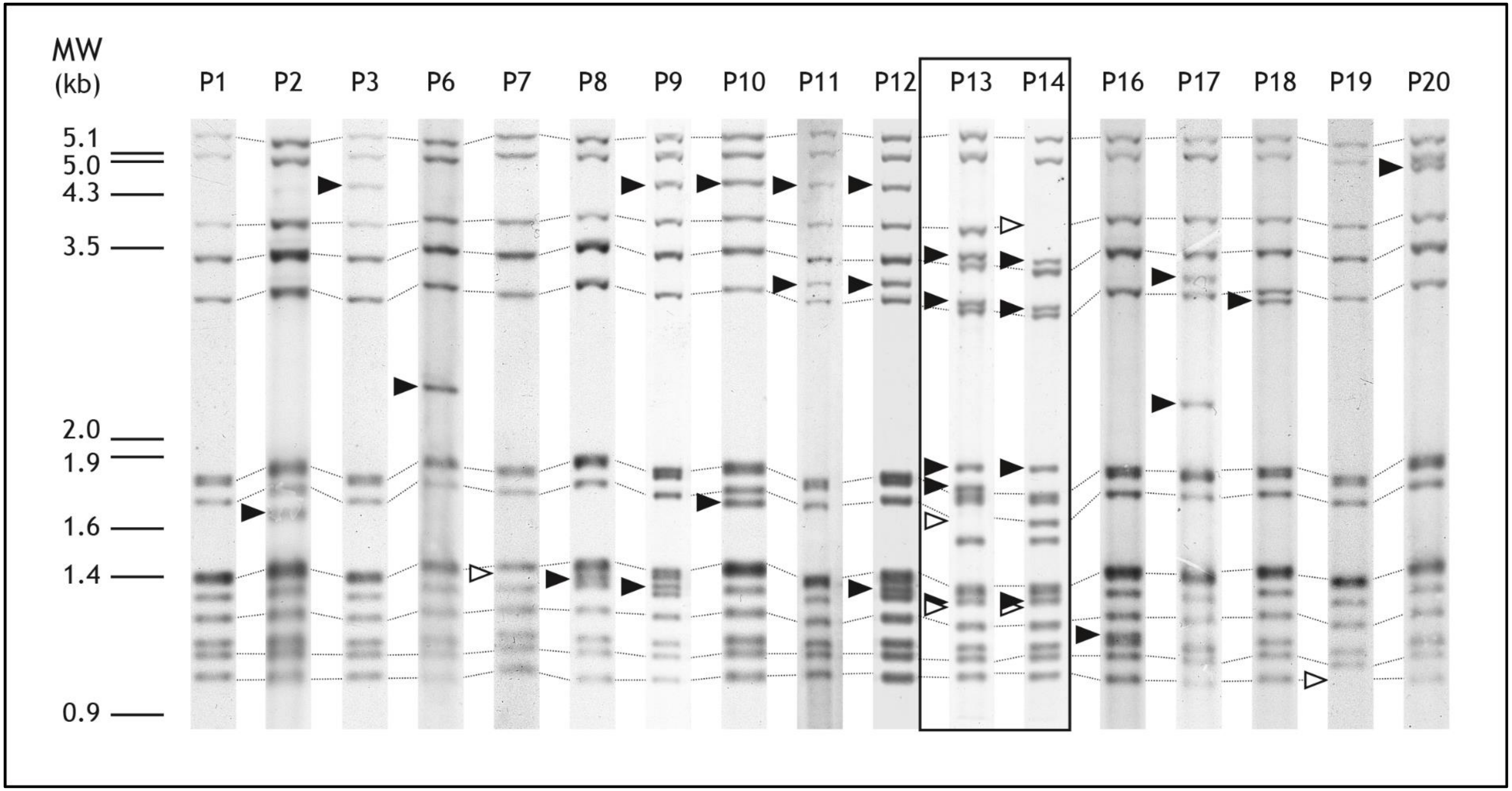
MW
(kb)
5.1
5.0
4.3
3.5
2.0
1.9
1.6
1.4
0.9
P1
P2
P3
P6
P7
P8
P9
P10
P11
P12
P13
P14
P16
P17
P18
P19
P20

(3b)

Figure S3. Overview of IS*900*-RFLP profiles of *Mycobacterium avium* subsp. *paratuberculosis* isolates from Germany after chromosomal DNA digestion with *Pst*I, (**3a**) as schematic representation and (**3b**) as images originating from different gels. A digoxigenin-labelled specific 453 bp fragment of IS*900* without digestion site for *Bst*EII or *Pst*I was used as probe for the Southern blots. The numbers above the lanes are the IS*900*-RFLP profile P designations. The arrowheads show the differences between profile P1 and the other types: (▶) additional or shifted bands or (▷) the absence of bands. Lane M (on the right and the left) shows the reference bands of molecular weight marker III (Roche Diagnostics GmbH, Mannheim, Germany). Corresponding bands in the profiles were connected with dotted lines in Figure S3b. All these profiles were identified for isolates from Germany in this study with the exception of profiles P4, P5, and P15 in Figure S3a.

Profiles P1-P4 were designated according to Whipple et al., 1990, [59] and Möbius et al., 2008, [52]; P5 was designated according to IS*900*-RFLP profile after *Pst*I digestion of a cattle isolate, (Figure 3, lane 1) in Saunders et al., 2003, [108]; profiles P6, P16 - P19 were designated according to Möbius et al., 2017, [29]; profiles P7 - P12 according to Möbius et al., 2008, [52]; the framed profiles P13 and P14 (intermediate profiles of S-type / subtype III strains) were published before by Möbius et al., 2009, [10]; profiles P15 and P16 are identical with PnewI and PnewIII in Sodoma et al., 2017, [51]; profile P20 is novel.

Table S1. *Mycobacterium avium* subsp. *paratuberculosis* (*Map*) isolates from cattle in Germany, their origin and respective genotyping results using IS*900*-RFLP, MIRU-VNTR- and SSR-analysis. A total of 438 isolates from 243 cattle herds were analyzed and assigned to 311 independent strains.

Isolate No [a]	Year	Origin State	Herd	Herd N°	Tissue	*Map* Type	IS*900*-RFLP *Bst*EII-*Pst*I [b]	MIRU-VNTR Code [c]	INMV [d]	SSR Profile [e]	Genotype combined	Isolate Reference
02A0239	2002	BW	BW-1	1	Intestine	C	C18-P16	32332228	2	7-4-4	66	-
02A1037	2002	BW	BW-2	2	Intestinal mucosa	C	C1-P1	32332228	2	7-4-4	4	-
03A0054	2003	BW	BW-3	3	Intestinal mucosa	C	C1-P1	32332228	2	7-4-4	4	-
03A0055	2003	BW	BW-3	3	Intestinal mucosa	C	C1-P1	32332228	2	7-4-4	4	-
04A2518	2004	BW	BW-4	4	Feces	C	C1-P3	42332128	19	7-4-4	37	-
04A2734	2004	BW	BW-5	5	Feces	C	C1-P1	32332128	6	≥11-4-5	16	-
05A0832	2005	BW	BW-6	6	Intestinal mucosa	C	C1-P3	42332128	19	7-4-4	37	-
05A1742	2005	BW	BW-7	7	Feces	C	C1-P1	32332228	2	7-4-4	4	-
05A1813	2005	BW	BW-8	8	Intestinal mucosa	C	C1-P1	42332228	1	7-4-4	1	-
05A2411	2005	BW	BW-9	9	Feces	C	C1-P1	32332228	2	7-4-4	4	-
05A2445	2005	BW	BW-10	10	Feces	C	C1-P1	32522228	33	7-4-4	22	-
05A3332	2005	BW	BW-11	11	Small intestine	C	C1-P1	22522228	12	7-4-4	18	-
08MA0556	2008	BW	BW-12	12	Feces	C	C1-P1	32332228	2	7-4-5	5	-
08MA0563	2008	BW	BW-12	12	Feces	C	C1-P1	32332228	2	7-4-5	5	-
08MA0569	2008	BW	BW-12	12	Feces	C	C1-P1	32332228	2	7-4-5	5	-
08MA0572	2008	BW	BW-12	12	Feces	C	C1-P1	32332228	2	7-4-5	5	-
09MA1377	2009	BW	BW-13	13	Feces	C	C1-P1	32522228	33	7-4-4	22	-
09MA1381	2009	BW	BW-13	13	Feces	C	C1-P1	32522228	33	7-4-4	22	-
n=18 / n=13												
07A0998	2007	BY	BY-1	14	Feces	C	C1-P1	32332228	2	7-4-4	4	-
07A0978	2007	BY	BY-2	15	Feces	S	I6-P13	42131(1.5)18	266	7-3-4	92	-
07A0986	2007	BY	BY-2	15	Feces	C	C41-P1	32332228	2	7-5-5	78	-
07A0988	2007	BY	BY-3	16	Feces	C	C17-P9	42332128	19	7-4-4	61	-
07A0989	2007	BY	BY-4	17	Feces	C	C1-P2	22332228	13	7-4-3	36	-
07A0992	2007	BY	BY-4	17	Feces	C	C17-P9	42332128	19	7-4-4	61	-
07A0990	2007	BY	BY-5	18	Feces	S	I6-P13	42131(1.5)18	266	7-3-4	92	-
07A0991	2007	BY	BY-5	18	Feces	C	C17-P9	42332128	19	7-4-4	61	-
07A0996	2007	BY	BY-6	19	Feces	C	C1-P1	42332228	1	7-4-5	2	-
07A0997	2007	BY	BY-6	19	Feces	C	C1-P1	42332228	1	7-4-5	2	-

07A0999	2007	BY	BY-7	20	Faeces	C	C1-P1	42332228	1	7-4-5	2	-
n=11 / n=10												
07A0969	2007	BB	BB-1	21	Feces	C	C17-P9	42332128	19	7-4-4	61	-
07A0970	2007	BB	BB-1	21	Feces	C	C17-P9	42332128	19	7-4-4	61	-
07A0971	2007	BB	BB-1	21	Feces	C	C17-P9	42332128	19	7-4-4	61	-
07A0972	2007	BB	BB-1	21	Feces	C	C41-P1	32332228	2	7-5-5	78	-
16MA0060	2016	BB	BB-2	22	Feces	C	C1-P1	22322226	262	7-4-4	33	-
16MA0062	2016	BB	BB-2	22	Feces	C	C1-P3	42332128	19	7-4-4	37	-
07A1074	2007	BB	BB-3	23	Intestinal LN	C	C24-P19	32522228	33	7-4-4	74	-
07A1132	2007	BB	BB-4	24	Intestinal LN	C	C1-P1	32332228	2	9-5-5	7	-
07A1133	2007	BB	BB-5	25	Intestinal LN	C	C1-P1	22522128	136	7-4-4	28	-
08MA0316	2008	BB	BB-6	26	Intestinal LN	C	C18-P16	42332228	1	7-4-5	64	-
08MA0317	2008	BB	BB-7	27	Intestinal LN	C	C1-P1	42332228	1	7-4-5	2	-
08MA0319	2008	BB	BB-8	28	Intestinal LN	C	C18-P16	42332228	1	7-4-5	64	-
08MA0394	2008	BB	BB-9	29	Intestinal LN	C	C18-P16	42332228	1	7-4-5	64	-
08MA0395	2008	BB	BB-10	30	Intestinal LN	C	C18-P16	42332228	1	7-4-5	64	-
08MA0403	2008	BB	BB-11	31	Intestinal LN	C	C1-P1	32332229	4	7-4-4	12	-
08MA0407	2008	BB	BB-12	32	Intestinal LN	C	C1-P1	32332228	2	≥11-5-5	9	-
08MA0587	2008	BB	BB-13	33	Intestinal LN	C	C1-P1	32522228	33	7-4-4	22	-
07A1023	2007	BB	BB-14	34	Intestinal LN	C	C1-P3	43332128	143	7-4-4	39	-
08MA0814	2008	BB	BB-15	35	Intestinal LN	C	C24-P19	32522228	33	7-4-4	74	-
n=19 / n=17												
08MA1340	2008	HE	HE-1	36	Feces	C	C17-P9	42332128	19	7-4-4	61	-
08MA1343	2008	HE	HE-2	37	Feces	C	C17-P8	42332228	1	7-4-4	56	-
08MA1344	2008	HE	HE-3	38	Feces	C	C1-P1	32332228	2	7-4-5	5	-
08MA1345	2008	HE	HE-3	38	Feces	C	C1-P1	32332228	2	7-4-4	4	-
08MA1346	2008	HE	HE-3	38	Feces	C	C1-P1	32332228	2	7-4-5	5	-
08MA1347	2008	HE	HE-4	39	Feces	C	C1-P3	42332128	19	7-4-4	37	-
08MA1348	2008	HE	HE-4	39	Feces	C	C1-P3	42332128	19	8-4-4	38	-
08MA1403	2008	HE	HE-5	40	Intestine	C	C1-P3	43332128	143	7-4-4	39	-
08MA1812	2008	HE	HE-5	40	Intestine	C	C1-P3	43332128	143	7-4-4	39	-
08MA1407	2008	HE	HE-6	41	Intestine	C	C1-P1	32332228	2	7-4-5	5	-
08MA1815	2008	HE	HE-7	42	Feces	C	C1-P1	32332228	2	≥11-5-5	9	-
09MA1221	2008	HE	HE-8	43	Boot swab	C	C1-P1	32332228	2	7-4-4	4	-
09MA1222	2008	HE	HE-9	44	Feces	C	C18-P16	32332228	2	10-5-5	67	-
09MA1223	2008	HE	HE-10	45	Feces	C	C1-P1	42332228	1	7-4-5	2	-

09MA1224	2008	HE	HE-11	46	Feces	C	C1-P3	43332128	143	7-4-4	39	-
09MA1226	2009	HE	HE-12	47	Feces	C	C1-P1	32332228	2	7-4-5	5	-
09MA1227	2009	HE	HE-13	48	Feces	C	C1-P3	42332128	19	7-4-4	37	-
09MA1229	2009	HE	HE-14	49	Boot swab	C	C1-P1	32322228	117	7-4-4	24	-
09MA1231	2009	HE	HE-14	49	Boot swab	C	C1-P3	43332128	143	7-4-4	39	-
12MA1689	2012	HE	HE-15	50	Boot swab	C	C1-P3	43332128	143	7-4-4	39	-
12MA1690	2012	HE	HE-16	51	Boot swab	C	C1-P1	22522226	80	7-4-4	23	-
12MA1691	2012	HE	HE-17	52	Boot swab	C	C18-P16	22332228	13	≥11-5-5	69	-
12MA1692	2012	HE	HE-18	53	Boot swab	C	C1-P3	43332128	143	7-4-4	39	-
12MA1693	2012	HE	HE-19	54	Boot swab	C	C1-P3	43332128	143	7-4-4	39	-
12MA1695	2012	HE	HE-20	55	Boot swab	C	C1-P1	32522228	33	7-4-4	22	-
n=25 / n=23												
07A0097	2006	MV	MV-1	56	Feces	C	C1-P1	32522228	33	7-4-4	22	-
07A0098	2006	MV	MV-2	57	Feces	C	C1-P1	32522228	33	7-4-4	22	-
07A0099	2006	MV	MV-2	57	Feces	C	C1-P1	32522228	33	7-4-4	22	-
07A0102	2006	MV	MV-3	58	Feces	C	C1-P1	42332228	1	7-4-4	1	-
07A0104	2006	MV	MV-4	59	Feces	C	C1-P1	32332228	2	7-4-5	5	-
07A0812	2007	MV	MV-4	59	Feces	C	C1-P1	32332228	2	7-4-4	4	-
07A0105	2006	MV	MV-5	60	Feces	C	C1-P1	42332228	1	7-4-4	1	-
07A0108	2006	MV	MV-6	61	Feces	C	C1-P1	32522228	33	7-4-4	22	-
07A0810	2007	MV	MV-7	62	Intestinal LN	C	C1-P1	32332218	3	7-4-4	11	-
08MA0539	2008	MV	MV-8	63	Feces	C	C1-P1	32522228	33	7-4-4	22	-
08MA0544	2008	MV	MV-8	63	Feces	C	C1-P1	32332228	2	7-4-4	4	-
08MA0542	2008	MV	MV-9	64	Feces	C	C1-P1	42332228	1	7-4-4	1	-
08MA0551	2008	MV	MV-9	64	Feces	C	C1-P1	42332228	1	7-4-4	1	-
08MA0543	2008	MV	MV-10	65	Feces	C	C1-P1	32522228	33	7-4-4	22	-
08MA0545	2008	MV	MV-11	66	Feces	C	C1-P1	32522228	33	7-4-4	22	-
08MA0548	2008	MV	MV-12	67	Feces	C	C1-P1	42332228	1	7-4-5	2	-
08MA0550	2008	MV	MV-13	68	Feces	C	C18-P1	32522228	33	7-4-4	63	-
07A0947	2007	MV	MV-14	69	Intestinal LN	C	C1-P1	32332228	2	7-4-4	4	-
n=18 / n=16												
05A3195	2003	NI	NI-1	70	Feces	C	C1-P1	22522226	80	7-4-4	23	-
05A3196	2004	NI	NI-1	70	Feces	C	C1-P1	22522226	80	7-4-4	23	-
05A3197	2004	NI	NI-1	70	Feces	C	C1-P1	22522226	80	7-4-4	23	-
05A3198	2004	NI	NI-1	70	Feces	C	C1-P1	22522226	80	7-4-4	23	-
05A3199	2004	NI	NI-1	70	Feces	C	C1-P1	22522226	80	7-4-4	23	-

05A3219	2004	NI	NI-1	70	Feces	C	C1-P1	22522226	80	7-4-4	23	-
05A3208	2004	NI	NI-2	71	Feces	C	C1-P1	22522228	12	7-4-4	18	-
06A0035	2005	NI	NI-3	72	Feces	C	C41-P7	32332228	2	7-5-5	80	-
06A0041	2005	NI	NI-4	73	Feces	C	C1-P1	42332128	19	7-4-4	20	-
06A0046	2005	NI	NI-5	74	Feces	C	C1-P1	32522228	33	7-4-4	22	-
06A0048	2005	NI	NI-5	74	Feces	C	C1-P1	32522228	33	7-4-4	22	-
06A0052	2005	NI	NI-5	74	Feces	C	C1-P1	32522228	33	7-4-4	22	-
06A0056	2005	NI	NI-6	75	Feces	C	C41-P7	32332228	2	7-5-5	80	-
06A0057	2005	NI	NI-6	75	Feces	C	C1-P1	22522226	80	7-4-4	23	-
06A1124	2005	NI	NI-7	76	Feces	C	C17-P9	12332228	248	7-4-4	62	-
06A1180	2006	NI	NI-7	76	Feces	C	C1-P1	32522228	33	7-4-4	22	-
06A1134	2006	NI	NI-8	77	Feces	C	C1-P1	42332128	19	7-4-4	20	-
06A1182	2006	NI	NI-9	78	Feces	C	C17-P9	42332228	1	7-4-4	60	-
06A1162	2006	NI	NI-10	79	Feces	C	C1-P1	22522228	12	7-4-4	18	-
06A1163	2006	NI	NI-10	79	Feces	C	C41-P1	32332128	6	7-5-5	79	-
06A1177	2006	NI	NI-11	80	Feces	C	C1-P1	22522226	80	7-4-4	23	-
n=21 / n=14												
06A0826	2006	NRW	NRW-1	81	Feces	C	C43-P3	42332128	19	7-4-4	83	-
06A0830	2006	NRW	NRW-1	81	Feces	C	C41-P7	32332228	2	7-5-5	80	[8]
06A1107	2005	NRW	NRW-1	81	Feces	C	C42-P3	42332128	19	7-4-4	82	[8]
06A1108	2005	NRW	NRW-2	82	Feces	C	C43-P3	42332128	19	7-4-4	83	[8]
05A2674	2005	NRW	NRW-2	82	Feces	C	C43-P3	42332128	19	7-4-4	83	[8]
06A1097	2005	NRW	NRW-3	83	Feces	C	C1-P1	32322228	117	7-4-4	24	-
06A1100	2005	NRW	NRW-3	83	Feces	C	C17-P8	42332228	1	7-4-4	56	[8]
06A1283 g	2006	NRW	NRW-3	83	Feces	C	C 17-P8	32332228	2	7-4-4	57	-
06A1284 g	2006	NRW	NRW-3	83	Feces	C	C41-P7	32332228	2	7-5-5	80	-
06A1263	2006	NRW	NRW-4	84	Feces	C	C17-P8	32332228	2	7-4-4	57	-
06A1264	2006	NRW	NRW-4	84	Feces	C	C17-P8	32332228	2	7-4-4	57	-
06A1265	2006	NRW	NRW-4	84	Feces	C	C17-P8	32332228	2	7-4-4	57	-
06A0990	2005	NRW	NRW-4	84	Feces	C	C17-P8	32332228	2	7-4-4	57	-
06A0991	2005	NRW	NRW-4	84	Feces	C	C17-P8	32332228	2	7-4-4	57	-
06A0992	2005	NRW	NRW-4	84	Feces	C	C17-P8	32332228	2	7-4-4	57	-
06A0993	2005	NRW	NRW-4	84	Feces	C	C17-P8	32332228	2	7-4-4	57	[8]
06A0994	2005	NRW	NRW-4	84	Feces	C	C17-P8	32332228	2	7-4-4	57	-
06A0995	2005	NRW	NRW-4	84	Feces	C	C17-P8	32332228	2	7-4-4	57	-
06A0996	2005	NRW	NRW-4	84	Feces	C	C17-P8	32332228	2	7-4-4	57	-

06A0997	2005	NRW	NRW-4	84	Feces	C	C17-P8	32332228	2	7-4-4	57	[8]
06A0998	2005	NRW	NRW-4	84	Feces	C	C1-P1	32332228	2	7-4-4	4	[8]
06A1268	2006	NRW	NRW-5	85	Feces	C	C1-P3	42332128	19	7-4-4	37	[8]
06A1270	2006	NRW	NRW-5	85	Feces	C	C17-P8	42332228	1	7-4-4	56	[8]
06A1271	2006	NRW	NRW-5	85	Feces	C	C1-P3	42332128	19	7-4-4	37	[8]
05A3269	2005	NRW	NRW-6	86	Feces	C	C1-P1	32332228	2	7-4-4	4	[8]
05A3271	2005	NRW	NRW-6	86	Feces	C	C1-P1	32332228	2	7-4-4	4	[8]
05A2669	2005	NRW	NRW-7	87	Feces	C	C1-P3	42332128	19	7-4-4	37	[8]
05A2670	2005	NRW	NRW-7	87	Feces	C	C1-P3	42332128	19	7-4-4	37	[8]
05A3258	2005	NRW	NRW-7	87	Mesenterial LN	C	C1-P10	42332128	19	7-4-4	42	[8]
05A3262	2005	NRW	NRW-7	87	Ileal mucosa	C	C41-P7	32332128	6	7-5-5	81	-
04A1381	2004	NRW	NRW-7	87	Feces	C	C1-P10	42332128	19	7-4-4	42	[8]
04A1382	2004	NRW	NRW-7	87	Feces	C	C17-P8	42332228	1	7-4-4	56	[8]
04A1383	2004	NRW	NRW-7	87	Feces	C	C1-P3	42332128	19	7-4-4	37	[8]
04A1384	2004	NRW	NRW-7	87	Feces	C	C1-P10	42332128	19	7-4-4	42	[8]
04A1396	2004	NRW	NRW-7	87	Feces	C	C22-P11	42332128	19	7-4-4	72	-
04A1387	2004	NRW	NRW-7	87	Feces	C	C1-P10	42332128	19	7-4-4	42	[8]
04A1398	2004	NRW	NRW-7	87	Feces	C	C17-P8	42332228	1	7-4-4	56	[8]
05A0158	2004	NRW	NRW-8	88	Feces	C	C1-P1	42332228	1	7-4-4	1	[8]
05A3263	2005	NRW	NRW-9	89	Feces	C	C1-P1	12322126	134	7-4-4	27	[8]
05A3265	2005	NRW	NRW-9	89	Feces	C	C1-P1	12522226	133	7-4-4	26	[8]
05A3267	2005	NRW	NRW-9	89	Feces	C	C1-P1	12322226	132	7-4-4	25	[8]
05A3268	2005	NRW	NRW-9	89	Feces	C	C1-P1	12322226	132	7-4-4	25	[8]
05A2760	2004	NRW	NRW-10	90	Feces	C	C41-P7	32332228	2	7-5-5	80	[8]
05A2763	2004	NRW	NRW-11	91	Feces	C	C1-P1	22522228	12	7-4-4	18	[8]
05A2765	2004	NRW	NRW-11	91	Feces	C	C1-P1	32322228	117	7-4-4	24	-
06A0147	2006	NRW	NRW-12	92	Feces	C	C1-P1	32332228	2	≥11-5-5	9	-
06A0153	2006	NRW	NRW-12	92	Feces	C	C1-(P1+P3)	(3+4)2332228	1 + 2	7-4/5-4/5	NA	-
06A0157	2006	NRW	NRW-12	92	Feces	C	C41-P7	32332228	2	7-5-5	80	-
06A0158	2006	NRW	NRW-12	92	Feces	C	C1-P3	42332128	19	7-4-4	37	-
06A0159	2006	NRW	NRW-12	92	Feces	C	C1-P1	32332228	2	≥11-5-5	9	-
06A0160	2006	NRW	NRW-12	92	Feces	C	C1-P1	32332228	2	≥11-5-5	9	-
06A0161	2006	NRW	NRW-12	92	Feces	C	C1-P1	32332228	2	≥11-5-5	9	-
06A0162	2006	NRW	NRW-12	92	Feces	C	C29-P3	42332128	19	7-4-4	77	-
06A0163	2006	NRW	NRW-12	92	Feces	C	C1-P1	32332228	2	≥11-5-5	9	-
06A0164	2006	NRW	NRW-12	92	Feces	C	C1-P3	42332128	19	7-4-4	37	-
06A0165	2006	NRW	NRW-12	92	Feces	C	C1-P1	32332228	2	≥11-5-5	9	-

06A0166	2006	NRW	NRW-12	92	Feces	C	C1-P1	32332228	2	≥11-5-5	9	-
06A0167	2006	NRW	NRW-12	92	Feces	C	C1-P1	42332228	1	7-4-4	1	-
06A0168	2006	NRW	NRW-12	92	Feces	C	C1-P1	32332228	2	≥11-5-5	9	-
06A0169	2006	NRW	NRW-12	92	Feces	C	C45-P1	32332228	2	≥11-5-5	86	-
06A0170	2006	NRW	NRW-12	92	Feces	C	C1-P1	42332228	1	7-4-4	1	-
06A0171	2006	NRW	NRW-12	92	Feces	C	C1-P3	42332128	19	7-4-4	37	-
06A0172	2006	NRW	NRW-12	92	Feces	C	C1-P3	42332128	19	7-4-4	37	-
06A0173	2006	NRW	NRW-12	92	Feces	C	C1-P1	32332228	2	10-5-5	8	-
06A0174	2006	NRW	NRW-12	92	Feces	C	C1-P1	32332228	2	≥11-5-5	9	-
06A0175	2006	NRW	NRW-12	92	Feces	C	C1-P1	32332228	2	≥11-5-6	10	-
06A0176	2006	NRW	NRW-12	92	Feces	C	C1-P1	32332228	2	≥11-5-5	9	-
06A0177	2006	NRW	NRW-12	92	Feces	C	C1-P1	32332228	2	≥11-5-5	9	-
06A1281 [f]	2006	NRW	NRW-13	93	Feces	C	C17-P9	42332128	19	7-4-4	61	-
06A1282 [f]	2006	NRW	NRW-13	93	Feces	C	C17-P8	42332228	1	7-4-4	56	-
n=70 / n=35												
10MA2461	2002	RP	RP-1	94	Feces	C	C17-P8	42332228	1	7-4-4	56	-
10MA2139	1996	RP	RP-2	95	Feces	C	C1-P1	32332128	6	≥11-4-5	16	-
10MA2561	2005	RP	RP-3	96	Feces	C	C18-P16	42332228	1	7-4-5	64	-
10MA2095	1995	RP	RP-4	97	Feces	C	C1-P3	42332128	19	7-4-4	37	-
10MA2392	2001	RP	RP-4	97	Feces	C	C1-P1	42332228	1	7-4-4	1	-
10MA2379	2000	RP	RP-5	98	Feces	C	C17-P8	42332228	1	7-4-4	56	-
10MA2590	2006	RP	RP-5	98	Intestinal content	C	C17-P8	42332228	1	7-4-4	56	-
10MA2090	1994	RP	RP-6	99	Feces	C	C1-P1	32332228	2	7-4-4	4	-
10MA2207	1997	RP	RP-7	100	Feces	C	C18-P16	42332228	1	7-4-5	64	-
10MA2497	2003	RP	RP-8	101	Feces	C	C1-P1	32332128	6	≥11-4-5	16	-
10MA2204	1997	RP	RP-9	102	Feces	C	C1-P1	32332228	2	7-4-4	4	-
10MA2337	1999	RP	RP-10	103	Feces	C	C1-P1	32332128	6	≥11-4-5	16	-
10MA2398	2001	RP	RP-11	104	Feces	C	C27-P1	42332228	1	7-4-5	75	-
10MA2064	1993	RP	RP-12	105	Feces	C	C1-P3	42332128	19	7-4-4	37	-
10MA2069	1994	RP	RP-12	105	Feces	C	C1-P3	42332128	19	7-4-4	37	-
10MA2083	1994	RP	RP-12	105	Feces	C	C1-P3	42332128	19	7-4-4	37	-
10MA2084	1994	RP	RP-12	105	Feces	C	C1-P3	42332128	19	7-4-4	37	-
10MA2087	1994	RP	RP-12	105	Feces	C	C1-P1	32522228	33	7-4-4	22	-
10MA2098	1995	RP	RP-12	105	Feces	C	C1-P3	42332128	19	7-4-4	37	-
10MA2135	1995	RP	RP-12	105	Feces	C	C1-P3	42332128	19	7-4-4	37	-
10MA2140	1996	RP	RP-12	105	Feces	C	C1-P3	42332128	19	7-4-4	37	-

10MA2514	2004	RP	RP-13	106	Feces	C	C1-P1	32332228	2	7-4-4	4	-
10MA2081	1994	RP	RP-14	107	Feces	C	C1-P3	42332128	19	7-4-4	37	-
10MA2086	1994	RP	RP-14	107	Feces	C	C1-P3	42332128	19	7-4-4	37	-
10MA2089	1994	RP	RP-14	107	Feces	C	C1-P3	42332128	19	7-4-4	37	-
10MA2099	1995	RP	RP-14	107	Feces	C	C27-P3	42332128	19	7-4-4	76	-
10MA2152	1996	RP	RP-14	107	Feces	C	C1-P3	42332128	19	7-4-4	37	-
10MA2516	2004	RP	RP-14	107	Feces	C	C1-P3	42332128	19	7-4-4	37	-
10MA2517	2004	RP	RP-14	107	Feces	C	C1-P3	42332128	19	7-4-4	37	-
10MA2341	1999	RP	RP-15	108	Feces	C	C1-P1	32332228	2	7-4-4	4	-
10MA2585	2005	RP	RP-15	108	Feces	C	C1-P1	32332228	2	7-4-4	4	-
10MA2586	2005	RP	RP-15	108	Feces	C	C1-P1	32332228	2	7-4-4	4	-
10MA2599	2006	RP	RP-15	108	Feces	C	C1-P1	32332228	2	7-4-4	4	-
10MA2462	2002	RP	RP-16	109	Feces	C	C1-P1	32332228	2	7-4-4	4	-
10MA2503	2004	RP	RP-17	110	Feces	C	C43-P3	42332128	19	7-4-4	83	-
10MA2415	2001	RP	RP-18	111	Feces	C	C1-P1	42332228	1	7-4-5	2	-
10MA2251	1998	RP	RP-19	112	Feces	C	C1-P2	32332228	2	7-4-3	35	-
10MA2383	2000	RP	RP-20	113	Feces	C	C1-P1	42332228	1	7-4-5	2	-
10MA2575	2005	RP	RP-20	113	Intestinal content	C	C1-P1	32522228	33	7-4-4	22	-
10MA2557	2005	RP	RP-21	114	Feces	C	C1-P1	32332228	2	7-4-4	4	-
10MA2306	1999	RP	RP-22	115	Feces	C	C1-P1	32332228	2	7-4-4	4	-
10MA2070	1994	RP	RP-23	116	Feces	C	C1-P3	42332128	19	7-4-4	37	-
10MA2174	1996	RP	RP-23	116	Feces	C	C1-P2	32332228	2	7-4-3	35	-
10MA2220	1997	RP	RP-23	116	Feces	C	C1-P2	32332228	2	7-4-3	35	-
10MA2223	1997	RP	RP-23	116	Faeces	C	C1-P2	32332228	2	7-4-3	35	-
10MA2315	1998	RP	RP-23	116	Intestinal content	C	C1-(P2 + P3)	(3+4)2332(1+2)28	2 + 19	7-4-3/4	35 + 37	-
10MA2329	1999	RP	RP-23	116	Feces	C	C1-P3	42332128	19	7-4-4	37	-
10MA2330	1999	RP	RP-23	116	Feces	C	C1-P3	42332128	19	7-4-4	37	-
10MA2331	1999	RP	RP-23	116	Feces	C	C1-P3	42332128	19	7-4-4	37	-
10MA2384	2000	RP	RP-24	117	Feces	C	C1-P1	32332228	2	7-4-4	4	-
10MA2463	2002	RP	RP-24	117	Feces	C	C1-P1	32332228	2	7-4-4	4	-
10MA2232	1997	RP	RP-25	118	Feces	C	C1-P1	42332228	1	7-4-5	2	-
10MA2562	2005	RP	RP-26	119	Feces	C	C1-P1	22522218	217	7-4-4	32	-
10MA2115	1995	RP	RP-27	120	Feces	C	C1-P1	22522128	136	7-4-4	28	-
10MA2530	2004	RP	RP-28	121	Feces	C	C18-P16	42332218	5	7-4-4	68	-
10MA2580	2005	RP	RP-28	121	Feces	C	C18-P16	42332218	5	7-4-4	68	-
10MA2075	1994	RP	RP-29	122	Feces	C	C1-P1	22322226	262	7-4-4	33	-
10MA2142	1996	RP	RP-30	123	Feces	C	C1-P1	42332228	1	7-4-5	2	-

10MA2506	2004	RP	RP-31	124	Intestinal content	C	C1-(P1 + P3)	42332(1+2)28	1 + 19	7-4-4/5	2 + 37	-
10MA2203	1997	RP	RP-32	125	Feces	C	C1-P1	42332228	1	7-4-5	2	-
10MA2369	2000	RP	RP-32	125	Feces	C	C27-P1	42332228	1	7-4-5	75	-
10MA2405	2001	RP	RP-32	125	Feces	C	C1-P1	42332228	1	7-4-5	2	-
10MA2425	2001	RP	RP-33	126	Feces	C	C1-P1	42332228	1	7-4-5	2	-
10MA2480	2003	RP	RP-33	126	Faeces	C	C1-P1	4233222(6+8)	263 + 1	7-4-5	34 + 2	-
10MA2173	1996	RP	RP-34	127	Feces	C	C17-P8	4233222(6+8)	263 + 1	7-4-4	58 + 56	-
10MA2199	1997	RP	RP-34	127	Feces	C	C17-P8	4233222(6+8)	263 + 1	7-4-4	58 + 56	-
10MA2360	2000	RP	RP-34	127	Feces	C	C17-P8	42332228	1	7-4-4	56	-
10MA2394	2001	RP	RP-35	128	Feces	C	C17-P8	42332228	1	7-4-4	56	-
10MA2412	2001	RP	RP-35	128	Feces	C	C17-P8	42332228	1	7-4-4	56	-
10MA2332	1999	RP	RP-36	129	Feces	C	C1-P1	42332228	1	7-4-5	2	-
n=70 / n=43												
10MA2171	1996	SL	SL-1	130	Feces	C	C1-P1	22522128	136	7-4-4	28	-
10MA2429	2001	SL	SL-2	131	Feces	C	C1-P1	32(3+5)22228	117 + 33	7-4-4	24 + 22	-
10MA2542	2005	SL	SL-3	132	Feces	C	C18-P16	42332228	1	7-4-5	64	-
10MA2489	2003	SL	SL-4	133	Feces	C	C1-P1	42332218	5	7-4-5	14	-
10MA2539	2005	SL	SL-5	134	Feces	C	C1-P1	22522128	136	7-4-4	28	-
10MA2459	2002	SL	SL-6	135	Feces	C	C1-P3	433321(1+2)8	264 + 143	7-4-4	40 + 39	-
n=6 / n=8												
09MA0966	2009	SN	SN-1	136	Feces	C	C1-P3	42332128	19	7-4-4	37	-
09MA0968	2009	SN	SN-1	136	Feces	C	C1-P3	42332128	19	7-4-4	37	-
09MA1269	2009	SN	SN-1	136	Feces	C	C1-P3	42332128	19	7-4-4	37	-
09MA1273	2009	SN	SN-1	136	Feces	C	C1-P3	42332128	19	7-4-4	37	-
09MA1162	2009	SN	SN-1	136	Feces	C	C10-P6	42332218	5	7-4-4	50	-
09MA1163	2009	SN	SN-1	136	Feces	C	C1-P1	32332128	6	≥11-4-5	16	-
09MA1164	2009	SN	SN-1	136	Feces	C	C1-P1	32332128	6	≥11-4-5	16	-
09MA0967	2009	SN	SN-2	137	Feces	C	C18-P16	32332228	2	6-4-4	65	-
09MA1275	2009	SN	SN-3	138	Feces	C	C1-P1	32332128	6	>11-4-5	16	-
10MA0116	2009	SN	SN-4	139	Feces	C	C1-P1	32332228	2	7-4-4	4	-
10MA0117	2009	SN	SN-4	139	Feces	C	C1-P1	32332228	2	7-4-4	4	-
10MA0118	2009	SN	SN-4	139	Feces	C	C1-P1	32332228	2	7-4-5	5	-
10MA0120	2009	SN	SN-5	140	Feces	C	C1-P1	32332128	6	≥11-4-5	16	-
10MA0121	2009	SN	SN-5	140	Feces	C	C1-P1	32332128	6	≥11-4-5	16	-
10MA0122	2009	SN	SN-6	141	Feces	C	C1-P3	42332128	19	7-4-4	37	-
10MA0123	2009	SN	SN-6	141	Feces	C	C1-P3	42332128	19	7-4-4	37	-

10MA0124	2009	SN	SN-7	142	Feces	C	C1-P1	32332228	2	7-4-4	4	-
11MA0012	2010	SN	SN-8	143	Feces	C	C1-P1	32332128	6	≥11-4-5	16	-
11MA0017	2010	SN	SN-8	143	Feces	C	C1-P1	42332228	1	7-4-4	1	-
11MA0018	2010	SN	SN-8	143	Feces	C	C1-P1	42332228	1	7-4-4	1	-
07A0948	2007	SN	SN-9	144	Intestinal LN	C	C1-P1	42332228	1	7-4-4	1	-
08MA0069	2008	SN	SN-9	144	Intestinal LN	C	C47-P1	42332228	1	7-4-4	88	-
07A1061	2007	SN	SN-10	145	Intestinal LN	C	C1-P3	42332128	19	7-4-4	37	-
08MA0068	2008	SN	SN-10	145	Intestinal LN	C	C1-P1	32332228	2	7-4-4	4	-
07A1004	2007	SN	SN-11	146	Intestinal LN	C	C1-P1	32332228	2	9-5-5	7	-
08MA0212	2008	SN	SN-11	146	Intestinal LN	C	C5-P1	32332228	2	≥11-5-5	44	-
07A0746	2007	SN	SN-12	147	Intestinal LN	C	C1-P1	32332228	2	7-4-5	5	-
07A0949	2007	SN	SN-13	148	Intestinal LN	C	C1-P1	32332228	2	7-4-4	4	-
07A0950	2007	SN	SN-14	149	Intestinal LN	C	C13-P18	32332227	191	7-4-4	54	-
07A1003	2007	SN	SN-15	150	Intestinal LN	C	C1-P1	32332228	2	≥11-5-5	9	-
07A1005	2007	SN	SN-16	151	Intestinal LN	C	C10-P6	42322228	141	7-4-4	51	-
07A1017	2007	SN	SN-17	152	Intestinal LN	C	C1-P1	32332128	6	>11-4-5	16	-
07A1018	2007	SN	SN-18	153	Intestinal LN	C	C1-P1	32522228	33	7-4-4	22	-
07A1021	2007	SN	SN-19	154	Intestinal LN	C	C1-P1	32332228	2	7-4-5	5	-
07A1069	2007	SN	SN-20	155	Intestinal LN	C	C1-P1	32332228	2	7-4-5	5	-
07A1075	2007	SN	SN-21	156	Intestinal LN	C	C24-P19	32522228	33	7-4-4	74	-
07A1105	2007	SN	SN-22	157	Intestinal LN	C	C1-P3	42332128	19	7-4-4	37	-
07A1122	2007	SN	SN-23	158	Intestinal LN	C	C1-P3	42332128	19	7-4-4	37	-
07A1130	2007	SN	SN-24	159	Intestinal LN	C	C1-P1	22332228	13	7-4-4	19	-
08MA0067	2008	SN	SN-25	160	Intestinal LN	C	C1-P1	32332228	2	7-4-4	4	-
08MA0070	2008	SN	SN-26	161	Intestinal LN	C	C1-P1	32522228	33	7-4-4	22	-
08MA0214	2008	SN	SN-27	162	Intestinal LN	C	C18-P16	22522226	80	7-4-4	70	-
08MA0406	2008	SN	SN-28	163	Intestinal LN	C	C18-P16	22522226	80	7-4-4	70	-
08MA0309	2008	SN	SN-29	164	Intestinal LN	C	C1-P3	42332128	19	7-4-4	37	-
08MA0576	2008	SN	SN-30	165	Intestinal LN	C	C1-P1	32332228	2	7-4-4	4	-
08MA0592	2008	SN	SN-31	166	Intestinal LN	C	C1-P1	42332228	1	7-4-4	1	-
08MA0812	2008	SN	SN-32	167	Intestinal LN	C	C24-P19	32522228	33	7-4-4	74	-
08MA0822	2008	SN	SN-33	168	Intestinal LN	C	C1-P1	32332228	2	7-4-4	4	-
07A1062	2007	SN	SN-34	169	Intestinal LN	C	C10-P6	42332228	1	7-4-4	49	-
n=49 / n=41												-
05A3231	2004	ST	ST-1	170	Feces	C	C1-P3	42332128	19	7-4-4	37	-
07A1110	2007	ST	ST-2	171	Feces	C	C1-P1	32522228	33	7-4-4	22	-

07A1113	2007	ST	ST-2	171	Feces	C	C1-P1	32332228	2	7-4-4	4	-
07A1114	2007	ST	ST-2	171	Feces	C	C1-P1	32522228	33	7-4-4	22	-
07A1111	2007	ST	ST-3	172	Feces	C	C1-P1	32332228	2	7-4-4	4	-
07A1116	2007	ST	ST-4	173	Feces	C	C1-P1	32522228	33	7-4-4	22	-
07A1117	2007	ST	ST-4	173	Feces	C	C1-P1	32522228	33	7-4-4	22	-
08MA1295	2008	ST	ST-5	174	Feces	C	C1-P1	42332228	1	7-4-4	1	-
08MA1296	2008	ST	ST-6	175	Feces	C	C44-P1	32522228	33	7-4-4	85	-
08MA1298	2008	ST	ST-7	176	Feces	C	C1-P1	32522228	33	7-4-4	22	-
08MA1299	2008	ST	ST-8	177	Feces	C	C1-P1	32332228	2	≥11-5-5	9	-
08MA1300	2008	ST	ST-9	178	Feces	C	C1-P1	32332228	2	7-4-4	4	-
08MA1301	2008	ST	ST-10	179	Feces	C	C1-P1	22522228	12	7-4-4	18	-
10MA2004	2010	ST	ST-11	180	Feces	C	C18-P16	32332228	2	10-5-5	67	-
10MA2005	2010	ST	ST-11	180	Feces	C	C1-P1	32332228	2	7-4-4	4	-
11MA0001	2011	ST	ST-12	181	Feces	C	C1-P1	42332228	1	7-4-4	1	-
07A0760	2007	ST	ST-13	182	Intestinal LN	C	C1-P1	32522228	33	7-4-4	22	-
08MA0260	2008	ST	ST-13	182	Intestinal LN	C	C46-P1	42332229	190	8-4-4	87	-
07A1131	2007	ST	ST-14	183	Intestinal LN	C	C49-P17	42332218	5	7-4-4	90	-
08MA0307	2008	ST	ST-14	183	Intestinal LN	C	C49-P17	42332218	5	7-4-4	90	-
07A1002	2007	ST	ST-15	184	Intestinal LN	C	C1-P1	32332228	2	7-4-4	4	-
07A1015	2007	ST	ST-16	185	Intestinal LN	C	C1-P1	42332228	1	7-4-4	1	-
07A1016	2007	ST	ST-17	186	Intestinal LN	C	C1-P3	42332128	19	7-4-4	37	-
07A1024	2007	ST	ST-18	187	Intestinal LN	C	C1-P1	42332218	5	7-4-4	13	-
08MA0125	2008	ST	ST-19	188	Intestinal LN	C	C46-P1	42332229	190	8-4-4	87	-
08MA0123	2008	ST	ST-20	189	Intestinal LN	C	C1-P1	32522228	33	7-4-4	22	-
08MA0397	2008	ST	ST-21	190	Intestinal LN	C	C1-P1	42332228	1	7-4-5	2	-
08MA0582	2008	ST	ST-22	191	Intestinal LN	C	C17-P8	42332228	1	7-4-4	56	-
08MA0617	2008	ST	ST-23	192	Intestinal LN	C	C44-P1	32332228	2	7-4-4	84	-
n = 29 / n=26												
01A0074	2001	TH	TH-1	193	Liver-LN	C	C17-P9	42332128	19	7-4-4	61	-
01A0157	2001	TH	TH-1	193	Liver-LN	C	C17-P9	42332128	19	7-4-4	61	-
01A0194	2001	TH	TH-1	193	Liver-LN	C	C17-P9	42332128	19	7-4-4	61	-
01A0342	2001	TH	TH-1	193	Ileocaecal LN	C	C17-P9	42332128	19	7-4-4	61	-
01A0360	2001	TH	TH-1	193	Ileocaecal LN	C	C17-P9	42332128	19	7-4-4	61	-
01A0432 [h]	2001	TH	TH-1	193	Jejunal Ln	C	C17-P9	42332128	19	7-4-4	61	-
01A0441 [h]	2001	TH	TH-1	193	Muscle	C	C1-P1	42332228	1	7-4-4	1	-
01A0478	2001	TH	TH-1	193	Jejunal LN	C	C17-P9	42332128	19	7-4-4	61	-

01A0534 [i]	2001	TH	TH-1	193	Jejunal LN	C	C1-P1	32332228	2	7-4-5	5	-
01A0535 [i]	2001	TH	TH-1	193	Jejunal LN	C	C17-P9	42332128	19	7-4-4	61	-
01A0643	2001	TH	TH-1	193	Jejunal LN	C	C17-P9	42332128	19	7-4-4	61	-
01A0671	2001	TH	TH-1	193	Ileocaecal LN	C	C17-P9	42332128	19	7-4-4	61	-
01A0993	2001	TH	TH-1	193	Milk	C	C17-P9	42332128	19	7-4-4	61	-
01A1127	2001	TH	TH-1	193	Jejunum	C	C17-P9	42332128	19	7-4-4	61	-
01A1146	2001	TH	TH-1	193	Ileocaecal LN	C	C17-P9	42332128	19	7-4-4	61	-
01A1257	2001	TH	TH-1	193	Ileum	C	C17-P9	42332128	19	7-4-4	61	-
02A0021	2002	TH	TH-1	193	Ileocecal LN	C	C17-P9	42332128	19	7-4-4	61	-
02A0088	2002	TH	TH-1	193	Jejunal LN	C	C17-P9	42332128	19	7-4-4	61	-
02A0097	2002	TH	TH-1	193	Jejunum	C	C1-P1	42332228	1	7-4-4	1	-
02A0060	2002	TH	TH-1	193	Ileum	C	C17-P9	42332128	19	7-4-4	61	-
02A0117	2002	TH	TH-1	193	Jejunal LN	C	C17-P9	42332128	19	7-4-4	61	-
02A0120	2002	TH	TH-1	193	Mammary gland	C	C17-P9	42332128	19	7-4-4	61	-
02A0658	2002	TH	TH-1	193	Ileocecal LN	C	C17-P9	42332128	19	7-4-4	61	-
02A1192 [j]	2002	TH	TH-1	193	Ileum	C	C17-P9	42332128	19	7-4-4	61	-
02A1194 [j]	2002	TH	TH-1	193	Caecum	C	C16-P12	42332128	19	7-4-4	55	-
03A0619	2003	TH	TH-1	193	Faeces	C	C17-P9	42332128	19	7-4-4	61	-
03A1961 [l]	2003	TH	TH-1	193	Ileocecal LN	C	C17-P9	42332128	19	7-4-4	61	[97]
03A2307	2003	TH	TH-1	193	Jejunum	C	C17-P9	42332128	19	7-4-4	61	-
04A0043	2004	TH	TH-2	194	Feces	C	C1-P1	32332228	2	7-4-5	5	-
04A0053	2004	TH	TH-2	194	Feces	C	C1-P1	32522228	33	7-4-4	22	-
04A0084	2004	TH	TH-2	194	Feces	C	C1-P1	32522228	33	7-4-4	22	-
04A0335	2004	TH	TH-2	194	Feces	C	C1-P1	32332228	2	7-4-5	5	-
04A0360	2004	TH	TH-2	194	Feces	C	C1-P1	32522228	33	7-4-4	22	-
04A0382	2004	TH	TH-2	194	Feces	C	C1-P1	32422128	142	7-4-4	31	-
11MA3510	2011	TH	TH-2	194	Feces	C	C1-P1	32332228	2	7-4-5	5	-
05A2327 [k]	2005	TH	TH-3	195	Ileum	C	C9-P2	42332228	1	7-4-4	47	-
05A2328 [k]	2005	TH	TH-3	195	Spleen LN	C	C9-P2	32332228	2	7-4-4	48	-
05A2334	2005	TH	TH-3	195	Feces	C	C10-P6	42332218	5	7-4-4	50	-
08MA0084	2008	TH	TH-3	195	Feces	C	C10-P6	42332218	5	7-4-4	50	-
05A2335	2005	TH	TH-4	196	Feces	C	C1-P1	32332228	2	7-4-4	4	-
05A2339	2005	TH	TH-5	197	Feces	C	C1-P1	32332228	2	7-4-4	4	-
05A2340	2005	TH	TH-5	197	Feces	C	C1-P1	32332228	2	7-4-4	4	-
06A0026	2005	TH	TH-5	197	Feces	C	C1-P1	32332228	2	7-4-4	4	-
05A2416	2005	TH	TH-6	198	Feces	C	C17-P8	42332228	1	7-4-4	56	-
05A2421	2005	TH	TH-7	199	Feces	C	C1-P1	32332228	2	7-4-4	4	-

05A2422	2005	TH	TH-8	200	Feces	C	C1-P1	32522228	33	7-4-4	22	-
05A2424	2005	TH	TH-9	201	Feces	C	C10-P17	42332218	5	7-4-4	52	-
05A3247	2005	TH	TH-10	202	Feces	C	C1-P1	22522228	12	7-4-4	18	-
05A3250	2005	TH	TH-11	203	Feces	C	C1-P1	22522228	12	7-4-4	18	-
06A0025	2005	TH	TH-12	204	Feces	C	C1-P1	32332228	2	7-4-4	4	-
06A0032	2005	TH	TH-13	205	Feces	C	C1-P1	32332228	2	7-4-4	4	-
06A0564	2005	TH	TH-14	206	Feces	C	C1-P1	32332228	2	7-4-5	5	-
06A0565	2005	TH	TH-15	207	Feces	C	C41-P7	32332228	2	7-5-5	80	-
06A0567	2005	TH	TH-16	208	Feces	C	C41-P7	32332228	2	7-5-5	80	-
06A0569	2005	TH	TH-17	209	Feces	C	C41-P7	32332228	2	7-5-5	80	-
06A0801	2005	TH	TH-18	210	Feces	C	C41-P7	32332228	2	7-5-5	80	-
06A1040	2006	TH	TH-19	211	Feces	C	C1-P1	32332228	2	≥11-5-5	9	-
06A1041	2006	TH	TH-20	212	Feces	C	C1-P1	32332228	2	7-4-4	4	-
07A0748	2007	TH	TH-21	213	Intestinal LN	C	C1-P1	325223*28	30*	7-4-4	21	-
07A0749	2007	TH	TH-21	213	Intestinal LN	C	C1-P1	325223*28	30*	7-4-4	21	-
07A0803	2007	TH	TH-21	213	Intestinal LN	C	C1-P1	42332228	1	7-4-4	1	-
07A0752	2007	TH	TH-22	214	Intestinal LN	C	C1-P1	42332128	19	7-4-4	20	-
07A0753	2007	TH	TH-22	214	Intestinal LN	C	C1-P1	42332128	19	7-4-4	20	-
07A0804	2007	TH	TH-22	214	Intestinal LN	C	C1-P1	42332128	19	7-4-4	20	-
08MA0262	2008	TH	TH-23	215	Intestinal LN	C	C48-P18	32522228	33	7-4-4	89	-
08MA0533	2008	TH	TH-23	215	Intestinal LN	C	C2-P18	32522228	33	7-4-4	43	-
08MA1024	2008	TH	TH-23	215	Intestinal LN	C	C2-P18	32522228	33	7-4-4	43	-
08MA0639	2008	TH	TH-23	215	Intestinal LN	C	C1-P1	32332228	2	7-5-5	6	-
08MA0109	2008	TH	TH-24	216	Intestinal LN	C	C1-P1	32332228	2	7-4-4	4	-
08MA0622	2008	TH	TH-24	216	Intestinal LN	C	C1-P1	32332228	2	7-4-4	4	-
08MA0098	2008	TH	TH-24	216	Intestinal LN	C	C1-P1	32332228	2	7-4-4	4	-
08MA0535	2008	TH	TH-24	216	Intestinal LN	C	C1-P1	42332228	1	7-4-5	2	-
08MA0099	2008	TH	TH-25	217	Intestinal LN	C	C1-P1	32332228	2	7-4-4	4	-
08MA0124	2008	TH	TH-25	217	Intestinal LN	C	C1-P1	22522228	12	7-4-4	18	-
07A0951	2007	TH	TH-26	218	Intestinal LN	C	C1-P1	32332228	2	7-4-4	4	-
07A1134	2007	TH	TH-27	219	Intestinal LN	C	C1-P1	32332228	2	7-4-4	4	-
08MA0066	2008	TH	TH-28	220	Intestinal LN	C	C12-P3	42332128	19	7-4-4	53	-
08MA0100	2008	TH	TH-29	221	Intestinal LN	C	C1-P1	32332228	2	7-4-4	4	-
08MA0126	2008	TH	TH-30	222	Intestinal LN	C	C46-P1	42332229	190	8-4-4	87	-
08MA0127	2008	TH	TH-31	223	Intestinal LN	C	C46-P1	42332229	190	8-4-4	87	-
08MA0619	2008	TH	TH-32	224	Intestinal LN	C	C1-P1	42332228	1	7-4-4	1	-
08MA0638	2008	TH	TH-33	225	Intestinal LN	C	C2-P18	32522228	33	7-4-4	43	-

07A0750	2007	TH	TH-34	226	Intestinal LN	C	C1-P1	42322228	141	7-4-4	30	-
08MA0410	2008	TH	TH-35	227	Intestinal LN	C	C1-P1	32332228	2	≥11-5-5	9	-
08MA0829	2008	TH	TH-36	228	Intestinal LN	C	C1-P1	32332218	3	7-4-4	11	-
08MA0830	2008	TH	TH-37	229	Intestinal LN	C	C1-P1	32332218	3	7-4-4	11	-
08MA1026	2008	TH	TH-38	230	Intestinal LN	C	C17-P8	42332228	1	7-4-4	56	-
07A0697	2007	TH	TH-39	231	Intestinal LN	C	C1-P1	32522228	33	7-4-4	22	-
07A0700	2007	TH	TH-40	232	Intestinal LN	C	C1-P1	32522228	33	7-4-4	22	-
13MA2076	2012	TH	TH-41	233	Slurry	C	C1-P1	32332228	2	7-4-4	4	-
12MA1601	2012	TH	TH-42	234	Boot swab	C	C1-P1	32332(5*+2)28	16* + 2	7-4-4	17 + 4	-
13MA2079	2012	TH	TH-42	234	Slurry	C	C1-P1	323325*28	16*	7-4-4	17	-
12MA1602	2012	TH	TH-43	235	Boot swab	C	C1-P1	32332(5*+2)28	16* + 2	7-4-4	17 + 4	-
13MA2080	2012	TH	TH-43	235	Slurry	C	C1-P1	323325*28	16*	7-4-4	17	-
12MA1613	2012	TH	TH-44	238	Boot swab	C	C1-P1	32332228	2	7-4-4	4	-
12MA1661	2012	TH	TH-45	237	Boot swab	C	C22-P20	22522228	12	7-4-4	73	-
12MA1667	2012	TH	TH-46	238	Boot swab	C	C1-P1	22522228	12	7-4-4	18	-
12MA1677	2012	TH	TH-47	239	Boot swab	C	C1-P3	42332128	19	7-4-4	37	-
12MA1685	2012	TH	TH-48	240	Boot swab	C	C1-P1	22522228	12	7-4-4	18	-
13MA0002	2012	TH	TH-49	241	Boot swab	C	C5-P8	42332228	1	7-4-4	46	-
13MA2125	2012	TH	TH-50	242	Slurry	C	C1-P1	22332228	13	7-4-4	19	-
13MA0004	2012	TH	TH-51	243	Boot swab	C	C1-P1	32332228	2	7-4-4	4	-
n=102 / n=65												

[a] number within the strain collection of the Friedrich-Loeffler-Institut, Jena (Germany); [b] IS900-RFLP-pattern after digestion with *Bst*EII and *Pst*I; [c] numerical code comprising the number of tandem repeats (TR) at MIRU-VNTR loci 292, X3, 25, 47, 3, 7, 10, and 32; [d] INMV profile according to the MAC-INMV-SSR database [78]; [e] number of short sequence repeats at locus1 [G], locus 8 [GGT], and locus 9 [TGC]; [f, g, h, i, j, k] - In each case two strains that originated from identical host animal but different tissues exhibiting different genotypes.; [l] 03A1961 is identical to JII-1961 (Accession No. NZ_CP022105.1., [97]); (1.5)—At MIRU-VNTR locus 7, in addition to one repeat an imperfect repeat with a length of 12 nucleotides (nt)—(CGTTCGGCGCGC)—was identified by sequencing between the regular flanking sequences, appearing like 1.5 repeats;

3*—At MIRU-VNTR locus 7, in addition to two repeats a 12 nt sequence of flanking region (CGTGCGGCGAAG) followed by an imperfect repeat with a length of 12 nt (GCCGTGAGAACA) out of perfect repeat (22 nt) was identified by sequencing. Amplicon size in the agarose gel appeared like three repeats and was highlighted with 3*.

5*—At MIRU-VNTR locus 7, in addition to three repeats, between repeat 2 and 3, an additional sequence region of 34 nt (CGTGCGGCGAAGGCTGGGCCGGCCCGAAAAGC) was identified by sequencing, belonging to an additional 56 nt region, described previously [82]. Amplicon size in the agarose gel appeared like almost five repeats, therefore highlighted with 5*; INMV 16* and 30* profile assignments were made according to amplicon sizes, despite of the changed repeat sequences at VNTR locus 7, explained in the main text.

Underlined—Only one isolate with same genotype per herd was counted as an independent strain; Individual black boxes—include strains originating from an individual herd in each case; Mixed isolates were considered as a mixture of two strains with different genotypes; LN–lymph node; BW—Baden-Wuerttemberg, BY—Bavaria, BB—Brandenburg,

HE—Hesse, MV—Mecklenburg-Western Pomerania, NI—Lower Saxony, NRW—North Rhine-Westphalia, RP—Rhineland-Palatinate, SL—Saarland, SN—Saxony, ST—Saxony-Anhalt, TH—Thuringia (federal states in Germany).

Table S2. *Mycobacterium avium* subsp. *paratuberculosis* (*Map*) isolates from different non-cattle hosts in Germany, their origin and respective genotyping results using IS*900*-RFLP, MIRU-VNTR- and SSR-analysis. A total of 46 isolates from 44 animals of 8 animal species and one human from 26 regional sources (localities) of 8 federal states were assigned to 32 independent strains.

Isolate No [a]	Year	Origin Host	State	Location	Tissue	*Map* Type	IS*900*-RFLP *Bst*EII-*Pst*I [b]	MIRU-VNTR Code [c]	INMV [d]	SSR Profile [e]	Genotype combined	Isolate Reference
09MA1247	2009	Goat	BW	Loc1	Feces	C	C1-P1	32332228	2	7-4-4	4	-
09MA1250	2009	Goat	BW	Loc1	Feces	C	C1-P1	32332228	2	7-4-4	4	-
10MA2754	2010	Barbary sheep	BE	Loc2	Ileocaecal LN	C	C1-P1	42332228	1	7-4-5	2	-
16MA0490	2016	Goat	BB	Loc3 [h]	Feces	C	C1-P1	22322226	262	7-4-4	33	-
16MA0492	2016	Goat	BB	Loc3 [h]	Feces	C	C1-P3	42332128	19	7-4-4	37	
08MA1338	2008	Human	HE	Loc4	Intest. biopsy	C	C1-P1	32332128	6	7-4-5	15	-
07A0955	2007	Sheep	NI	Loc5	Intestine	S	-	22331(1.5) [i]18	220	-	-	-
07A1025	2007	Sheep	NI	Loc5	Ileum	S	-	22331(1.5)18	220	-	-	-
07A1027	2007	Sheep	NI	Loc5	Ileum	S	-	22331(1.5)18	220	-	-	-
14MA1119	2014	Sheep	NI	Loc5	Ileum	S	-	22331(1.5)18	220	7-3-4	-	-
14MA2048	2014	Sheep	NI	Loc5	Intestine	S	I2-P14	22331(1.5)18	220	7-3-4	91	-
09MA0079	2009	Sheep	NI	Loc6	Intestine	C	C17-P9	42332128	19	7-4-4	61	-
10MA1707	2010	Sheep	NI	Loc7	Colon	C	C1-P1	32522228	33	7-4-4	22	-
12MA0864	2012	Goat	NI	Loc8	Ileocaecal LN	C	C1-P1	32522228	33	7-4-4	22	-
12MA1039	2012	Goat	NI	Loc8	Intestine	C	C1-P1	32522228	33	7-4-4	22	-
12MA1214	2012	Goat	NI	Loc8	Jejunum	C	C1-P1	32522228	33	7-4-4	22	-
15MA0743	2015	Eur. bison	NI	Loc9	Feces	C	C41-P7	32332228	2	7-5-5	80	-
03A1310	2002	Sheep	NRW	Loc10	Mesenteric LN	S	I7-P14	52331(1.5)18	267	7-3-3	93	[10]
07A0762	2006	Sheep	NRW	Loc10	Ileum	S	-	52331(1.5)18	267	7-3-3	-	-
03A2230	2003	Sheep	NRW	Loc10	Mesenteric LN	C	C41-P7	32332228	2	7-5-5	80	-
04A0386 [f]	2003	Sheep	NRW	Loc11	Ileal mucosa	S	I6-P13	42131(1.5)18	266	7-3-4	92	[10]
05A0159	2004	Red deer	NRW	Loc12	Intest. mucosa	C	C1-P3	42332128	19	7-4-4	37	[8]
06A0820	2006	Red deer	NRW	Loc12	Intestine	C	C1-P3	42332128	19	7-4-4	37	[8]
06A0821	2006	Red deer	NRW	Loc12	Intestine	C	C41-P7	32332228	2	7-5-5	80	[8]

06A0822	2006	Red deer	NRW	Loc12	Intestine	C	C41-P7	32332228	2	7-5-5	80	[8]
06A0813	2006	Red deer	NRW	Loc13	Intestine	C	C17-P8	42342228	265	7-4-4	59	[8]
06A0814	2006	Red deer	NRW	Loc13	Intestine	C	C22-P1	32332228	2	7-4-4	71	[8]
06A0815	2006	Red deer	NRW	Loc13	Intestine	C	C41-P7	32332228	2	7-5-5	80	[8]
06A0816	2006	Red deer	NRW	Loc13	Intestine	C	C17-P8	32332228	2	7-4-4	57	[8]
06A0817	2006	Red deer	NRW	Loc13	Intestine	C	C17-P8	32332228	2	7-4-4	57	[8]
06A0818	2006	Red deer	NRW	Loc13	Intestine	C	C41-P7	32332228	2	7-5-5	80	[8]
06A0819	2006	Red deer	NRW	Loc13	Intestine	C	C17-P8	32332228	2	7-4-4	57	[8]
06A0987	2005	Goat	NRW	Loc14	Intestine	C	C1-P1	42332228	1	8-4-5	3	-
06A0988	2005	Sheep	NRW	Loc15	Feces	C	C1-P1	32332228	2	7-4-4	4	-
06A0989	2005	goat	NRW	Loc16	Intestine	C	C1-P1	42332228	1	7-4-4	1	-
06A1278 [g]	2006	Roe deer	NRW	Loc17	Feces	C	C5-P7	32332228	2	7-5-5	45	-
06A1279 [g]	2006	Roe deer	NRW	Loc17	Feces	C	C1-P7	32332228	2	7-5-5	41	-
09MA0432	2009	Goat	NRW	Loc18	Intestine	C	C1-P1	32332228	2	7-4-4	4	-
11MA0247	2011	Goat	NRW	Loc19	Intestinal LN	C	C1-P1	32332228	2	7-4-4	4	-
10MA1033	2010	Goat	NRW	Loc20	Ileum	C	C1-P1	32522228	33	7-4-4	22	-
11MA1887	2011	Fallow deer	SN	Loc21	Intestine	C	C18-P16	32332228	2	6-4-4	65	-
11MA0021	2011	Min. donkey	SN	Loc22	Feces	C	C1-P1	52332228	139	9-4-5	29	[94]
10MA1574	2010	Sheep	TH	Loc23	Feces	C	C1-P1	32332228	2	7-4-5	5	-
11MA0986	2011	Sheep	TH	Loc24	Intestinal LN	S	I6-P13	42131(1.5)18	266	7-3-4	92	-
12MA0282	2010	Fallow deer	TH	Loc25	Intestine	C	C1-P1	42332228	1	7-4-5	2	-
12MA0283	2011	Fallow deer	TH	Loc25	Intestine	C	C1-P1	42332228	1	7-4-5	2	-

[a] number within the strain collection of the Friedrich-Loeffler-Institut, Jena (Germany; [b] IS900-RFLP-pattern after digestion with *Bst*EII and *Pst*I; [c] numerical code comprising the number of tandem repeats (TR) at MIRU-VNTR loci 292, X3, 25, 47, 3, 7, 10, and 32; [d] INMV profile according to the MAC-INMV-SSR database (http://mac-inmv.tours.inra.fr), [78]; [e] number of short sequence repeats at locus1 [G], locus 8 [GGT], and locus 9 [TGC]; [f] 04A0386 is identical with JIII-386 (Accession No. NZ_CP042454, [31]); [g] isolates originated from the faeces of the same animal, but were cultured on different media exhibiting different genotypes; [h] location 3 is identical with cattle farm BB-2 in Brandenburg and cattle farm no. 22 in Germany, listed in Table S1; (1.5) [i] – At VNTR locus 7, in addition to the regularly repeat and flanking sequences an imperfect repeat with a length of 12 nt (CGTTCGGCGCGC) was identified; Underlined — only one isolate with same genotype per source was counted as an independent strain; Individual black boxes include strains originating from an individual source/location in each case; Eur. – European; Min. – miniature; LN–lymph node; Instest.– intestinal; BW—Baden-Wurttemberg, BE—Berlin, BB—Brandenburg, HE—Hesse, NI—Lower Saxony, NRW—North Rhine-Westphalia, SN—Saxony, TH—Thuringia

Table S3. Genotyping results of 63 *Mycobacterium avium* subsp. *paratuberculosis* (*Map*) isolates from different tissues of 22 individual animals

**Animal N°**	**Host**	**Origin Herd / Loc** [a]	**Isolate** [b] **Designation**	**Tissue origin**	***Map* type**	**IS*900*-RFLP *Bst*EII - *Pst*I** [c]	**MIRU-VNTR Code** [d]	**INMV** [e]	**SSR Profile** [f]	**Genotype combined**	**Reference**
1	cattle	TH-1	01A0432 [g]	Jejunal LN	C	C17-P9	42332128	19	7-4-4	61	-
			01A0435	Liver	C	C17-P9	42332128	19	7-4-4	61	-
			01A0441 [g]	Muscle	C	**C1-P1**	**42332228**	**1**	7-4-4	**1**	-
2	cattle	TH-1	01A0534 [g]	Jejunal LN	C	C1-P1	32332228	2	7-4-5	5	-
			01A0535 [g]	Jejunal LN	C	**C17-P9**	**42332128**	**19**	**7-4-4**	**61**	-
3	cattle	TH-1	02A0112	Feces	C	C17-P9	42332128	19	7-4-4	61	-
			02A0116	Ileocaecal LN	C	C17-P9	42332128	19	**7-4-5**	different	-
			02A0117 [g]	Jejunal LN	C	C17-P9	42332128	19	7-4-4	61	-
			02A0118	Liver	C	C17-P9	42332128	19	7-4-4	61	-
			02A0119	Liver LN	C	C17-P9	42332128	19	7-4-4	61	-
4	cattle	TH-1	02A1191	Feces	C	C17-P9	42332128	19	7-4-4	61	-
			02A1192 [g]	Ileum	C	C17-P9	42332128	19	7-4-4	61	-
			02A1194 [g]	Caecum	C	**C16-P12**	42332128	19	7-4-4	**55**	-
5	cattle	TH-3	05A2326	Feces rectum	C	C9-P2	42332228	1	7-4-4	47	-
			05A2327 [g]	Ileum LN	C	C9-P2	42332228	1	7-4-4	47	-
			05A2328 [g]	Spleen LN	C	C9-P2	**32332228**	**2**	7-4-4	**48**	-
			05A2330	Lung of foetus	C	C9-P2	42332228	1	7-4-4	47	-
6	cattle	NRW-3	06A1283 [g]	Feces 1	C	C 17-P8	32332228	2	7-4-4	57	-
			06A1284 [g]	Feces 2	C	**C41-P7**	32332228	2	**7-5-5**	**80**	-
7	cattle	NRW-13	06A1281 [g]	Feces 1	C	C 17-P9	42332128	19	7-4-4	61	-
			06A1282 [g]	Feces 2	C	**C 17-P8**	**42332228**	**1**	7-4-4	**56**	-
8	Roe deer	NRW-Loc17	06A1278 [h]	Feces 1	C	C5-P7	32332228	2	7-5-5	45	-
			06A1279 [h]	Feces 2	C	**C1-P7**	32332228	2	7-5-5	**41**	-
9	sheep	TH-Loc24	11MA0986 [h]	Mesenteric LN	S	I6-P13	421311*18	266	7-3-4	92	-
			11MA0987_1	Feces 1 (LJ)	S	I6-P13	421311*18	266	7-3-4	92	-

			11MA0987_2	Feces 2 (Her)	C	n.d.	**32332228**	**2**	**7-5-5**	different	-
10	cattle	HE-5	08MA1402	Feces	C	C1-P3	43332128	143	7-4-4	39	-
			08MA1403 [g]	Intestinal Mucosa	C	C1-P3	43332128	143	7-4-4	39	-
			08MA1404	Mesenteric LN	C	C1-P3	43332128	143	7-4-4	39	-
			08MA1405	Milk	C	C1-P3	43332128	143	7-4-4	39	-
11	cattle	HE-5	08MA1811	Feces	C	C1-P3	42332128	19	7-4-4	38	-
			08MA1812 [g]	Intestinal Mucosa	C	C1-P3	42332128	19	7-4-4	38	-
			08MA1813	Mesenteric LN	C	C1-P3	42332128	19	7-4-4	38	-
12	cattle	HE-6	08MA1406	Feces	C	C1-P1	32332228	2	7-4-5	5	-
			08MA1407 [g]	Intestinal Mucosa	C	C1-P1	32332228	2	7-4-5	5	-
			08MA1408	Mesenteric LN	C	C1-P1	32332228	2	7-4-5	5	-
			08MA1409	Milk	C	C1-P1	32332228	2	7-4-5	5	-
13	cattle	HE-7	08MA1814	Feces	C	C1-P1	32332228	2	≥11-5-5	9	-
			08MA1815 [g]	Intestinal Mucosa	C	C1-P1	32332228	2	≥11-5-5	9	-
			08MA1816	Mesenteric LN	C	C1-P1	32332228	2	≥11-5-5	9	-
14	cattle	MV-12	08MA0548 [g]	Feces	C	C1-P1	42332228	1	7-4-5	2	-
			08MA0549	Intestinal Mucosa	C	C1-P1	42332228	1	7-4-5	2	-
15	cattle	TH-1	01A0069	Ileum	C	C17-P9	42332128	19	7-4-4	61	-
			01A0074 [g]	Liver LN	C	C17-P9	42332128	19	7-4-4	61	-
			01A0075	Udder	C	C17-P9	42332128	19	7-4-4	61	-
16	cattle	TH-1	01A0192	Ileocaecal LN	C	C17-P9	42332128	19	7-4-4	61	-
			01A0194 [g]	Liver LN	C	C17-P9	42332128	19	7-4-4	61	-
17	cattle	TH-1	01A0476	Ileocaecal LN	C	C17-P9	42332128	19	7-4-4	61	-
			01A0478 [g]	Jejunal LN	C	C17-P9	42332128	19	7-4-4	61	-
18	cattle	TH-1	01A0642	Ileocecal LN	C	C17-P9	42332128	19	7-4-4	61	-
			01A0643 [g]	Jejunal LN	C	C17-P9	42332128	19	7-4-4	61	-
19	cattle	TH-1	03A2306	Ileum	C	C17-P9	42332128	19	7-4-4	61	-
			03A2307 [g]	Jejunum	C	C17-P9	42332128	19	7-4-4	61	-
			03A2308	Ileocaecal LN	C	C17-P9	42332128	19	7-4-4	61	-

20	bison	NI-Loc9	15MA0739	Valva ileocaecalis	C	n.d.	32332228	2	7-5-5	identical	-
			15MA0740	Ileum	C	n.d.	32332228	2	7-5-5	identical	-
			15MA0741	Ileocecal LN	C	n.d.	32332228	2	7-5-5	identical	-
			15MA0742	Jejunum	C	n.d.	32332228	2	7-5-5	identical	-
			15MA0743 [h]	Feces	C	C41-P7	32332228	2	7-5-5	identical	-
21	sheep	NRW-Loc10	03A1310 [h]	Mesenteric LN	C	I7-P14	523311*18	267	7-3-3	93	[10]
			03A1311	Ileocecal LN	C	I7-P14	523311*18	267	7-3-3	93	[10]
22	goat	NRW-Loc19	11MA0246	Intestine	C	C1-P1	32332228	2	7-4-4	4	-
			11MA0247 [h]	Intestinal LN	C	C1-P1	32332228	2	7-4-4	4	-

[a] N° of herd or regional origin (state-location) with regard to Table S1 and S2; [b] designation within the strain collection of the NRL for Paratuberculosis at Friedrich-Loeffler-Institut, Jena (Germany); [c] IS*900*-RFLP pattern after digestion with BstEII and PstI; [d] Number of tandem repeats (TR) at MIRU-VNTR loci 292, X3, 25, 47, 3, 7, 10, and 32; [e] INMV - INRA Nouzilly MIRU-VNTR (INMV) nomenclature for MIRU-VNTR patterns according to the MAC-INMV-SSR database (http://mac-inmv.tours.inra.fr), [78]; [f] number of short sequence repeats at loci 1 [G], 8 [GGT], 9 [TGC], (SSR1, SSR8, SSR9); [g, h] strains, which were also included in strain panels listed in Table S1 (g) and Table S2 (h); 1*—At VNTR locus 7, in addition to the regular repeat and flanking sequences an imperfect repeat with a length of 12 nt (CGTTCGGCGCGC) was identified by sequencing; LN—lymph node; (LJ)—cultivated in Löwenstein-Jensen medium; (Her)—cultivated in Herrolds Egg Yolk Medium; In bold—different genotype in comparison to genotypes of other isolate(s) from the same animal; Feces 1 or 2—isolates originated from the same animal and sampling time, but were cultivated on different media; n.d.—not detected.

Table S4. List of different profiles and discriminatory index (DI) of IS*900*-RFLP, MIRU-VNTR and SSR typing, alone and in combination (A), and Summary (B). The DI was calculated according to Hunter and Gaston [114].

$$DI = 1 - \left[\frac{1}{N(N-1)} \sum_{j=1}^{S} n_j(n_j - 1) \right]$$

N - Total number of unrelated / independent strains in the respective typing scheme, *S* - Total number of distinct profiles discriminated, nj - Number of unrelated / independent strains belonging to the jth profile

Table S4 (A). List of different profiles and discriminatory index (DI) of different typing methods

Typing Method	N	*S* (n profiles)	profiles	nj (number / profile)	DI
RFLP *Bst*EII	303	26	C1	202	0.544
			C2	2	
			C5	3	
			C9	1	
			C10	5	
			C12	1	
			C13	1	
			C16	1	
			C17	22	
			C18	17	
			C22	3	
			C24	4	
			C27	3	
			C29	1	
			C41	17	
			C42	1	
			C43	3	
			C44	2	
			C45	1	
			C46	4	
			C47	1	
			C48	1	
			C49	1	
			I2	1	
			I6	4	
			I7	1	
RFLP *Pst*I	302	17	P1	184	0.607
			P2	4	
			P3	36	
			P6	4	
			P7	15	
			P8	15	
			P9	10	
			P10	1	

			P11	1	
			P12	1	
			P13	4	
			P14	2	
			P16	16	
			P17	2	
			P18	3	
			P19	4	
			P20	1	
RFLP *Bst*EII + *Pst*I	313	39	C1-P1	174	0.674
			C1-P2	5	
			C1-P3	31	
			C1-P7	1	
			C1-P10	1	
			C2-P18	2	
			C5-P1	1	
			C5-P7	1	
			C5-P8	1	
			C9-P2	1	
			C10-P6	4	
			C10-P17	1	
			C12-P3	1	
			C13-P18	1	
			C16-P12	1	
			C17-P8	14	
			C17-P9	10	
			C18-P1	1	
			C18-P16	16	
			C22-P1	1	
			C22-P11	1	
			C22-P20	1	
			C24-P19	4	
			C27-P1	2	
			C27-P3	1	
			C29-P3	1	
			C41-P1	3	
			C41-P7	14	
			C42-P3	1	
			C43-P3	3	
			C44-P1	2	
			C45-P1	1	
			C46-P1	4	
			C47-P1	1	
			C48-P18	1	
			C49-P17	1	
			I2-P14	1	
			I6-P13	4	
			I7-P14	1	
MIRU-VNTR	318	33	1	58	0.847

			2	95	
			3	3	
			4	1	
			5	7	
			6	12	
			12	11	
			13	4	
			16*	2	
			19	38	
			30*	1	
			33	37	
			80	6	
			117	4	
			132	1	
			133	1	
			134	1	
			136	4	
			139	1	
			141	2	
			142	1	
			143	8	
			190	4	
			191	1	
			217	1	
			220	1	
			248	1	
			262	3	
			263	2	
			264	1	
			265	1	
			266	4	
			267	1	
SSR (Loci 1, 8, 9)	299	15	6-4-4	2	0.54
			7-3-3	1	
			7-3-4	5	
			7-4-3	3	
			7-4-4	198	
			7-4-5	39	
			7-5-5	20	
			8-4-4	5	
			8-4-5	1	
			9-4-5	1	
			9-5-5	2	
			10-5-5	3	
			≥11-4-5	7	
			≥11-5-5	7	
			≥11-5-6	1	
RFLP + MIRU-VNTR + SSR	343	93	1	17	0.954
			2	16	

3	1
4	52
5	13
6	1
7	2
8	1
9	7
10	1
11	3
12	1
13	1
14	1
15	1
16	9
17	2
18	10
19	2
20	3
21	1
22	29
23	4
24	4
25	1
26	1
27	1
28	4
29	1
30	1
31	1
32	1
33	3
34	1
35	2
36	1
37	23
38	1
39	8
40	1
41	1
42	1
43	2
44	1
45	1
46	1
47	1
48	1
49	1
50	2
51	1
52	1

53	1
54	1
55	1
56	12
57	3
58	1
59	1
60	1
61	8
62	1
63	1
64	7
65	2
66	1
67	2
68	1
69	1
70	2
71	1
72	1
73	1
74	4
75	2
76	1
77	1
78	2
79	1
80	14
81	1
82	1
83	3
84	1
85	1
86	1
87	4
88	1
89	1
90	1
91	1
92	4
93	1

Table S4 (B). Summary: Discriminatory index (DI) of IS*900*-RFLP, MIRU-VNTR and SSR typing (this study)

**Typing Method**		
IS*900*-RFLP *Bst*EII	No.[1]	303
	DI	0.544
IS*900*-RFLP *Pst*I	No.	302
	DI	0.607
IS*900*-RFLP (*Bst*EII + *Pst*I)	No.	313
	DI	0.674
MIRU-VNTR	No.	318
	DI	0.847
SSR	No.	299
	DI	0.54
IS*900* RFLP + MIRU-VNTR + SSR	No.	343
	DI	0.954

[1] number of unrelated / independent strains

Table S5. Number of cattle herds and isolates investigated in different federal states of Germany and the respective number of different genotypes (GT). A total of 438 isolates in 243 herds were tested. In addition, the number of GTs found in non-cattle host species are listed.

**Federal State**	**Cattle herd [n]**	**Cattle isolates [n]**	**GT cattle [n]**	**Non-cattle**	**GT non-cattle [n]**
BW	13	18	8	Goat	1*
BY	7	11	6	-	-
BE	-	-	-	Barbary sheep	1
BB	15	19	13	Goat	2*
HE	20	25	14	Human	1
MV	14	18	7	-	-
NI	11	21	8	Goat,	1*
NI	-	-	-	Sheep	2 + 1*
NI	-	-	-	Bison	1
NRW	13	70	22	Sheep	2 + 2*
NRW	-	-	-	Deer	4 + 3*
NRW	-	-	-	Goat	2 + 2*
RP	36	70	18	-	-
SL	6	6	7	-	-
SN	34	49	18	Deer	1*
SN	-	-	-	Donkey	1
ST	23	29	14	-	-
TH	51	102	30	Sheep	1 + 1*
TH	-	-	-	Deer	1

* number of GTs found also in cattle; BB—Brandenburg, BE—Berlin, BW—Baden-Wuerttemberg, BY—Bavaria, HE—Hesse, MV—Mecklenburg-Western Pomerania, NI—Lower Saxony, NRW—North Rhine-Westphalia, RP—Rhineland Palatinate, SL—Saarland, SN—Saxony, ST—Saxony-Anhalt, TH—Thuringia

Table S6. List of the combined genotypes (GT) of *Mycobacterium avium* subsp. *paratuberculosis* identified in this study and their origin

GT N°	States [n]	Cattle Herd [n] [a]	States [n]	Non-cattle Host Host	Strains [n] [b]	States
1 [c]	7	16	7	goat	1	NRW [d]
2 [c]	8	15	7	sheep, deer	2	BE, TH [d]
3	1	-	-	goat	1	NRW
4 [c]	9	48	9	goat, sheep	4	BW [d], NRW [d]
5	5	12	5	sheep	1	TH [d]
6	1	1	1	-	-	-
7	2	2	2	-	-	-
8	1	1	1	-	-	-
9	6	7	6	-	-	-
10	1	1	1	-	-	-
11	2	3	2	-	-	-
12	1	1	1	-	-	-
13	1	1	1	-	-	-
14	1	1	1	-	-	-
15	1	-	-	human	1	HE
16	3	9	3	-	-	-
17	1	2	1	-	-	-
18	5	10	5	-	-	-
19	2	2	2	-	-	-
20	2	3	2	-	-	-
21	1	1	1	-	-	-
22 [c]	11	26	10	goat, sheep	3	NI [d], NRW
23	2	4	2	-	-	-
24	3	4	3	-	-	-
25	1	1	1	-	-	-
26	1	1	1	-	-	-
27	1	1	1	-	-	-
28	3	4	3	-	-	-
29	1	-	-	donkey	1	SN
30	1	1	1	-	-	-
31	1	1	1	-	-	-
32	1	1	1	-	-	-
33	2	2	2	goat	1	BB [d]
34	1	1	1	-	-	-
35	1	2	1	-	-	-
36	1	1	1	-	-	-
37 [c]	8	21	8	deer	2	BB [d], NRW [d]
38	1	1	1	-	-	-
39	3	8	3	-	-	-
40	1	1	1	-	-	-
41	1	-	-	deer	1	NRW
42	1	1	1	-	-	-
43	1	2	1	-	-	-
44	1	1	1	-	-	-
45	1	-	-	deer	1	NRW

46	1	1	1	-	-	-
47	1	1	1	-	-	-
48	1	1	1	-	-	-
49	1	1	1	-	-	-
50	2	2	2	-	-	-
51	1	1	1	-	-	-
52	1	1	1	-	-	-
53	1	1	1	-	-	-
54	1	1	1	-	-	-
55	1	1	1	-	-	-
56	5	12	5	-	-	-
57	1	2	1	deer	1	NRW [d]
58	1	1	1	-	-	-
59	1	-	-	deer	1	NRW
60	1	1	1	-	-	-
61	6	7	5	sheep	1	NI
62	1	1	1	-	-	-
63	1	1	1	-	-	-
64	3	7	3	-	-	-
65	1	1	1	deer	1	SN [d]
66	1	1	1	-	-	-
67	3	7	3	-	-	-
68	1	1	1	-	-	-
69	1	1	1	-	-	-
70	1	2	1	-	-	-
71	1	-	-	deer	1	NRW
72	1	1	1	-	-	-
73	1	1	1	-	-	-
74	2	4	2	-	-	-
75	1	2	1	-	-	-
76	1	1	1	-	-	-
77	1	1	1	-	-	-
78	2	2	2	-	-	-
79	1	1	1	-	-	-
80	3	10	3	deer, sheep, bison	3	NRW [d]
81	1	1	1	-	-	-
82	1	1	1	-	-	-
83	2	3	2	-	-	-
84	1	1	1	-	-	-
85	1	1	1	-	-	-
86	1	1	1	-	-	-
87	2	4	2	-	-	-
88	1	1	1	-	-	-
89	1	1	1	-	-	-
90	1	1	1	-	-	-
91	1	-	-	sheep	1	NI
92	3	2	1	sheep	2	NRW, TH
93	1	-	-	sheep	1	NRW

[a] number [n] of cattle herds corresponds with the number of independent cattle strains; [b] number of independent strains; [c] predominant combined genotypes (GTs) in German cattle: GT1, GT2, GT4, GT22, GT37; [d] In this federal

state the respective GT was isolated from both cattle and non-cattle hosts; BB—Brandenburg, BE—Berlin, BW—Baden-Wuerttemberg, HE—Hesse, NI—Lower Saxony, NRW—North Rhine-Westphalia, SN—Saxony, TH—Thuringia;

Table S7. *Mycobacterium avium* subsp. *paratuberculosis* genotypes (GT) of non-cattle strains, their distribution across federal states with non-cattle and cattle origin.

GT	Host	Strain [a] [n]	Origin	Origin of cattle strains
1 [b]	Goat	1	NRW	BW, MV, NRW, RP, SN, ST, TH
2 [b]	Fallow Deer, Barbary Sheep	2	BE, TH	BY, BB, HE, MV, RP, ST, TH
3	Goat	1	NRW	-
4 [b]	Goat, Sheep	4	BW, NRW	BW, BY, HE, MV, NRW, RP, SN, ST, TH
5	Sheep	1	TH	BW, HE, MV, SN, TH
15	Human	1	HE	-
22 [b]	Sheep, Goat	3	NI, NRW	BW, BB, HE, MV, NI, RP, SL, SN, ST, TH
29	Miniature Donkey	1	SN	-
33	Goat	1	BB	BB, RP
37 [b]	Goat, Red Deer	2	BB, NRW	BW, BB, HE, NRW, RP, SN, ST, TH
41	Roe Deer	1	NRW	-
45	Roe Deer	1	NRW	-
57	Red Deer	1	NRW	NRW
59	Red Deer	1	NRW	-
61	Sheep	1	NI	BY, BB, HE, NRW, TH
65	Fallow Deer	1	SN	SN
71	Red Deer	1	NRW	-
80	Sheep, Red Deer	3	NRW	NI, NRW, TH
91	Sheep	1	NI	-
92	Sheep	2	NRW, TH	BY
93	Sheep	1	NRW	-

[a] independent strains of non-cattle hosts; [b] predominant GTs in cattle in Germany; BB—Brandenburg, BE—Berlin, BW—Baden-Wuerttemberg, BY—Bavaria, HE—Hesse, MV—Mecklenburg-Western Pomerania, NI—Lower Saxony, NRW—North Rhine-Westphalia, RP—Rhineland-Palatinate, SL—Saarland, SN—Saxony, ST—Saxony-Anhalt, TH—Thuringia

Table S8. Intra-herd diversity of *Mycobacterium avium* subsp. *paratuberculosis* and the number and type of target region(s) with polymorphisms. IS*900*-RFLP profiles after *Bst*EII and *Pst*I digestion, MIRU-VNTRs at 8 loci (VNTR 292, X3, 25, 47, 3, 7, 10, and 32), and SSR at 3 Loci (SSR 1, 8, 9) belong to the 13 target regions used for genotyping. Two or more isolates (with same or different year of isolation) were examined from 70 out of 243 cattle herds, for a total of 267 isolates. In 48 of these herds more than one genotype (GT) was detected.

State	Herd	Isolates [n] [a]	GT [n]	Target R diff [n]	RFLP-Profile / Target region that differed
BW	BW-3	2 (2003)	1	0	-
	BW-12	4 (2008)	1	0	-
	BW-13	2 (2009)	1	0	-
BY	BY-2	2 (2007)	2	9	RFLP (*Bst*EII, *Pst*I); VNTR292, 25, 3, 7, 10; SSR8, 9
	BY-4	2 (2007)	2	5	RFLP (*Bst*EII, *Pst*I); VNTR292, 7; SSR9
	BY-5	2 (2007)	2	7	RFLP (*Bst*EII, *Pst*I); VNTR25, 3, 7, 10; SSR8

	BY-6	2 (2007)	1	0	-
BB	BB-1	4 (2007)	2	6	RFLP (*Bst*EII, *Pst*I); VNTR25, 7; SSR8, 9
	BB-2	2 (2016)	2	4	RFLP (*Pst*I); VNTR292, 7, 32
HE	HE-3	3 (2008)	2	1*	SSR9
	HE-4	2 (2008)	2	1*	SSR1
	HE-5	2 (2008)	1	0	-
	HE-14	2 (2009)	2	5	RFLP (*Pst*I); VNTR292, X3, 47, 7
MV	MV-2	2 (2007)	1	0	-
	MV-4	2 (2006/07)	2	1*	SSR9
	MV-8	2 (2008)	2	2	VNTR25 and 47
	MV-9	2 (2008)	1	0	-
NI	NI-1	6 (2004/04)	1	0	-
	NI-5	3 (2005)	1	0	-
	NI-6	2 (2005)	2	8	RFLP (*Bst*EII, P*st*I), VNTR292, 25, 47, 32; SSR8, 9
	NI-7	2 (2005/06)	2	5	RFLP (*Bst*EII, *Pst*I), VNTR292, 25, 47
	NI-10	2 (2006)	2	7	RFLP (*Bst*EII), VNTR292, 25, 47, 7; SSR8, 9
NRW	NRW-1	3 (2005/06)	3	6	RFLP (*Bst*EII, PstI), VNTR292, 7; SSR8, 9
	NRW-2	2 (2005)	1	0	-
	NRW-3	4 (2005/06)	4	6	RFLP (*Bst*EII, *Pst*I), VNTR292, 47; SSR8, 9
	NRW-4	12 (2005/06)	2	2	RFLP (*Bst*EII, *Pst*I)
	NRW-5	3 (2006)	2	3	RFLP (*Bst*EII, *Pst*I); VNTR7
	NRW-6	2 (2005)	1	0	-
	NRW-7	11 (2004/05)	5	6	RFLP (*Bst*EII, *Pst*I), VNTR292, 7; SSR8, 9
	NRW-9	4 (2005)	3	2	VNTR25 and 7
	NRW-11	2 (2005)	2	1*	VNTR292
	NRW-12	23 (2006)	8	7	RFLP (*Bst*EII, *Pst*I), VNTR292, 7; SSR1, 8, 9
	NRW-13	2 (2006)	2	2	RFLP (*Pst*I), VNTR7
RP	RP-4	2 (1995/2001)	2	2	RFLP (*Pst*I), VNTR7
	RP-5	2 (2000/06)	1	0	-
	RP-12	8 (1993/94/95)	2	5	RFLP (*Pst*I), VNTR292, 25, 47, 7
	RP-14	7 (1994/95/96/2004)	2	1 (3)	RFLP (*BstE*II), 3 bands different
	RP-15	4 (1999/2005/06)	1	0	-
	RP-20	2 (2000/05)	2	4	VNTR292, 25, and 47, SSR9
	RP-23	8 (1994/96/97/98/99)	2	4	RFLP (*Pst*I), VNTR292, 7; SSR9
	RP-24	2 (2000/02)	1	0	-
	RP-28	2 (2004/05)	1	0	-
	RP-32	3 (1997/2000/01)	2	1 (3)	RFLP (*Bst*EII), 3 bands different
	RP-33	2 (2001/03)	2	1*	VNTR32
	RP-34	3 (1996/97/2000)	2	1*	VNTR32
	RP-35	2 (2001)	1	0	-
SL	SL-2	1 (2001)	Mix (2)	1*	VNTR25
	SL-6	1 (2002)	Mix (2)	1*	VNTR10
SN	SN-1	7 (2009)	3	7	RFLP (*Bst*EII, *Pst*I), VNTR292, 7, 10; SSR1, 9
	SN-4	3 (2009)	2	1*	SSR9
	SN-5	2 (2009)	1	0	-
	SN-6	2 (2009)	1	0	-
	SN-8	3 (2010)	2	4	VNTR292 and 7, SSR1, 9
	SN-9	2 (2007/08)	2	1*	RFLP (*Bst*EII), 1 band different
	SN-10	2 (2007/08)	2	3	RFLP (*Pst*I), VNTR292, 7
	SN-11	2 (2007/08)	2	2	RFLP (*Bst*EII), SSR1
ST	ST-2	3 (2007)	2	2	VNTR25, 47
	ST-4	2 (2007)	1	0	NA
	ST-11	2 (2010)	2	5	RFLP (*Bst*EII, PstI), SSR1, 8, 9
	ST-13	2 (2007/08)	2	6	RFLP (*Bst*EII), VNTR292, 25, 47, 32; SSR1
	ST-14	2 (2007/08)	1	0	-
TH	TH-1	28 (2001/02/03)	4	5	RFLP (*Bst*EII, *Pst*I), VNTR 292, 7; SSR9
	TH-2	7 (2004/11)	3	4	VNTR25, 47, 7; SSR9
	TH-3	4 (2005/08)	3	4	RFLP (*Bst*EII, *Pst*I); VNTR292, 10
	TH-5	3 (2005/06)	1	0	-
	TH-21	3 (2007)	2	4	VNTR292, 25, 47, 7

TH-22	3 (2007)	1	0	-
TH-23	4 (2008)	3	6	RFLP (*Bst*EII, *Pst*I); SSR9
TH-24	4 (2008)	2	2	VNTR292, SSR9
TH-25	2 (2008)	2	3	VNTR292, 25, 47
TH-42	2 (2012)	2	1*	VNTR7
TH-43	2 (2012)	2	1*	VNTR7

[a] year(s) of strain isolation in individual herds is given in brackets; BW—Baden-Wuertemberg, BY—Bavaria, BE—Berlin, BB—Brandenburg, HE—Hesse, MV—Mecklenburg-Western Pomerania, NI—Lower Saxony, NRW—North Rhine-Westphalia, RP—Rhineland-Palatinate, SL—Saarland, SN—Saxony, ST—Saxony-Anhalt, TH—Thuringia; Target R diff [n]—number of targets (genetic markers) that showed differences in isolates of this herd

Table S9: Different genotype diversity in cattle farms indicated by Shannon diversity index and Simpson index.

Federal State	Farm	Genotypes	Isolates [n]	H	D
NRW	NRW-12	GT9	11		
		GT37	4		
		GT1	2		
		GT8	1		
		GT10	1		
		GT77	1		
		GT80	1		
		GT86	1		
			Σ 23	1.57	0.268
TH	TH-1	GT61	24		
		GT1	2		
		GT5	1		
		GT55	1		
			Σ 28	0.56	0.733

H—Shannon diversity index H = -Σpi * ln (pi), higher values indicate greater diversity (Spellerberg and Fedor, 2003, doi:10.1046/j.1466-822X.2003.00015.x); D—Simpson index D = Σni (ni-1) / N (N-1), the value for Simpson's Index D ranges between 0 and 1, the higher the value, the lower the diversity (Simpson, 1949, doi:10.1038/163688a0); NRW—North Rhine-Westphalia, TH—Thuringia
